# Expression and Function of Connexin 43 in Human Gingival Wound Healing and Fibroblasts

**DOI:** 10.1371/journal.pone.0115524

**Published:** 2015-01-13

**Authors:** Rana Tarzemany, Guoqiao Jiang, Hannu Larjava, Lari Häkkinen

**Affiliations:** Department of Oral Biological and Medical Sciences, Faculty of Dentistry, University of British Columbia, Vancouver, Canada; Albert Einstein College of Medicine, UNITED STATES

## Abstract

Connexins (C×s) are a family of transmembrane proteins that form hemichannels and gap junctions (GJs) on the cell membranes, and transfer small signaling molecules between the cytoplasm and extracellular space and between connecting cells, respectively. Among C×s, suppressing C×43 expression or function promotes skin wound closure and granulation tissue formation, and may alleviate scarring, but the mechanisms are not well understood. Oral mucosal gingiva is characterized by faster wound closure and scarless wound healing outcome as compared to skin wounds. Therefore, we hypothesized that C×43 function is down regulated during human gingival wound healing, which in fibroblasts promotes expression of genes conducive for fast and scarless wound healing. Cultured gingival fibroblasts expressed C×43 as their major connexin. Immunostaining of unwounded human gingiva showed that C×43 was abundantly present in the epithelium, and in connective tissue formed large C×43 plaques in fibroblasts. At the early stages of wound healing, C×43 was strongly down regulated in wound epithelial cells and fibroblasts, returning to the level of normal tissue by day 60 post-wounding. Blocking of C×43 function by C×43 mimetic peptide Gap27 suppressed GJ-mediated dye transfer, promoted migration, and caused significant changes in the expression of wound healing-associated genes in gingival fibroblasts. In particular, out of 54 genes analyzed, several MMPs and TGF-β1, involved in regulation of inflammation and extracellular matrix (ECM) turnover, and VEGF-A, involved in angiogenesis, were significantly upregulated while pro-fibrotic ECM molecules, including Collagen type I, and cell contractility-related molecules were significantly down regulated. These responses involved MAPK, GSK3α/β and TGF-β signaling pathways, and AP1 and SP1 transcription factors. Thus, suppressed function of C×43 in fibroblasts promotes their migration, and regulates expression of wound healing-associated genes via AP1, SP1, MAPK, GSK3α/β and TGF-β signaling pathways, and may promote fast and scarless wound healing in human gingiva.

## Introduction

Connexins (C×s) are a family of transmembrane proteins that assemble to form connexons (hemichannels) or gap junctions (GJs). Each connexin protein is composed of four transmembrane-spanning domains, two extracellular loops, and the cytoplasmic domains including N-terminus, C-terminal domain and the cytoplasmic loop. Assembly of six connexin subunits generates one connexon or hemichannel, which functions in autocrine and paracrine signaling by providing a pathway for transfer of signaling molecules, including ATP, NAD^+^, Ca^2+^ and glutamate, between cells and extracellular environment. Two connexons from neighboring cells can also dock to form GJs, which provide conduits for direct exchange of small (<1 kDa) signaling molecules between communicating cells [1–3]. In addition to the channel functions, connexins participate in intracellular signaling cascades, and regulate gene expression and cell migration [2,4–6].

Connexins are expressed virtually by all cells in the body, and play crucial role during development and normal tissue function, and contribute to development of various pathologies [5,6]. In addition, they may play a role in skin wound healing [7–9]. In skin, expression and localization of connexins has been best described in epithelium of normal tissue and in epithelium of experimental wounds in various murine and human models. For instance, in normal skin in mice, epithelial cells at various layers, and cultured human skin keratinocytes, express several connexins, including C×26, C×30, C×30.3, C×31, C×31.1, C×37, C×40 and C×43 [2,10–13]. Likewise, based on immunostaining, human epidermis contains at least C×26, C×30 and C×43 [14]. Interestingly, wound healing induces rapid but transient changes in epithelial cell connexins. For instance, in mouse skin, C×26, C×30, C×31, C×31.1 and C×43 are strongly down regulated in the migrating wound epithelium, while C×26 and C×30 are upregulated at the wound margins [10–13]. Until re-epithelialization is complete, expression of connexins is further spatiotemporally regulated at different epithelial layers [11]. Similar findings have also been reported for C×26, C×30 and C×43 in human skin wound epithelium [14,15]. During early stages of murine skin wound healing, decreased expression of C×26 and C×43 in hair follicles at the wound site, and upregulation of C×43 in blood vessels close to wound area has also been reported [11], but very little is known about expression of connexins in wound fibroblasts.

In general, fibroblasts in normal skin express connexins, and appear connected to each other by GJs [16–19]. The major GJ protein in cultured murine skin fibroblasts is C×43 [20]. Similarly, cultured human skin fibroblasts express C×43 as their major connexin, but they also express lower levels of C×40 and C×45 [2,21]. Electron microscopy analysis has suggested that wound myofibroblast-like cells are connected to each other by GJs [22], but the identity of the connexins involved and their spatiotemporal regulation during wound healing is unclear.

Based on the above studies, C×43 appears a key connexin expressed by skin cells, and it is strongly down regulated in the wound epithelium at the early stages of wound healing. To study its role in more detail, different strategies to further suppress its function or expression in experimental murine skin wounds have been used. For instance, transient blocking of C×43 function at the early stage of wound healing by ACT1, a peptide that binds to the cytoplasmic carboxy-tail of C×43 [23], or transient suppressing its expression by topical C×43-specific antisense oligodeoxynucleotides (AS-ODN), promotes re-epithelialization and wound closure via increased keratinocyte migration and proliferation [10,11]. Studies from conditional C×43 knockout mice have also shown an earlier onset of keratinocyte migration and increased proliferation, resulting in faster skin wound closure as compared to control mice [12,13]. In addition to the epithelial effects, transient treatment of murine skin wounds with ACT1, C×43 AS-ODN or siRNA suppresses inflammation, and promotes certain aspects of connective tissue healing. For instance, connective tissue cell proliferation, angiogenesis, collagen deposition, and earlier myofibroblast recruitment and wound contraction are stimulated, resulting to reduced wound connective tissue size at the early remodeling stage as compared to control wounds [10–13,24–30]. Thus, down regulation of C×43 appears to accelerate wound granulation tissue formation and remodeling. Interestingly, transient down-regulation of C×43 expression by AS-ODN or modulation of its function by ACT1 at the very early stage of wound healing also improves the clinical appearance of the wounds and increases wound breaking strength in long term in mouse and pig models [30,31]. However, it is unclear whether the above effects of early and transient C×43 inhibition in wounds are secondary to the reduced inflammation and/or due to altered C×43 function in fibroblasts. In any case, down regulation of C×43 by AS-ODN, or blocking of its function by ACT1, or by Gap26 and Gap27, two C×43 mimetic peptides that block its hemichannel and GJ functions [3], promotes fibroblast, and also keratinocyte, proliferation and migration *in vitro* [2,12,20,24,25,32,33]. Thus, connexin inhibition may also have direct wound healing promoting effects on these cells, but the mechanisms remain largely undefined.

Interestingly, wound healing in human and pig oral mucosal attached gingiva is faster and results in significantly reduced scar formation as compared to similar skin wounds [34–37]. Therefore, gingival wound healing provides a model to study molecular and cellular pathways that regulate fast and scarless wound healing. Given that connexins play a role in wound healing, it is possible that they have a key role determining the wound healing outcome also in human gingiva. Previously, connexins have been localized in oral mucosal wounds in a mouse buccal (cheek) mucosal wound model. In the unwounded buccal mucosal epithelium, a non-keratinized epithelium distinct from keratinized gingival epithelium, keratinocytes expressed C×26, C×40 and C×43, and their levels were significantly elevated as compared to epidermis in the same animals [38]. Similar to skin wounds, these connexins were down regulated in migrating keratinocytes in the mucosal wounds [38]. Connexin expression has also been studied in normal human gingival epithelium, where keratinocytes express C×26 and C×43 [39–41]. However, nothing is known about connexin expression and function in gingival connective tissue cells and during gingival wound healing. Therefore, the aim of the present study was to characterize in detail the localization and function of C×43, a key connexin associated with skin wound healing, in the fast and scarless human gingival wound healing. We hypothesized that C×43 function is down regulated during human gingival wound healing, which in fibroblasts promotes expression of genes conducive for fast and scarless wound healing.

## Materials and Methods

### Tissue Samples

Tissue sections from experimental wounds created in palatal attached gingival mucosa in three healthy males (mean age 38 years) that have been previously extensively characterized were used [35,42–46]. Briefly, identical, standardized, full-thickness excisional wounds (about 12 mm long, 2 mm wide and at least 10 mm away from each other) were prepared under local anesthesia in the palatal mucosa in an area between the canine and the third molar using a double-bladed scalpel. The tissue obtained from the initial wounds served as the control samples (day 0 sample). After the surgery, subjects were instructed to use standard dosages of acetaminophen or ibuprofen for postoperative pain control. Wound biopsies were collected at days 3, 7, 14, 28, and 60 after wounding. Immediately after collection, the samples were embedded in Optimal Cutting Temperature Compound (Sakura Finetek Inc., Torrance, CA, USA) and frozen in liquid nitrogen. Tissue sections (6 μm) were cut using a 2800 Frigocut Cryostat Microtome (Leica, Nussloch, Germany), placed on 3-aminopropyltriethoxysilane-coated slides and stored at −86°C until use. For the study, minimum of three tissue/mid-wound sections from two to three subjects at each time point were used.

### Cell Culture

Four gingival fibroblast lines (GFBLs) were isolated from clinically healthy attached gingiva from healthy human donors, as previously described [47] (S1 Table). Cells were routinely maintained in Dulbecco’s Modified Eagle’s medium (DMEM), supplemented with 1% antibiotic/antimycotic and 10% fetal bovine serum (FBS) (Gibco Life Technologies, Inc., Grand Island, NY, USA) at 37°C and 5% CO_2_. Cells were routinely seeded for experiments when they reached about 95% confluence. Experiments were performed at passages 5 to 10.

### Ethics Statement

Gingival tissue donors provided a written informed consent, and procedures were reviewed and approved by the Office of Research Ethics of the University of British Columbia, and complies with the ethical rules for human experimentation that are stated in the 1975 Declaration of Helsinki.

### Blocking of C×43 Function by Mimetic Peptides or MFA

To block the C×43 function, GFBLs were seeded on 6-well plates (42,000 cells/cm^2^) in their normal growth medium. At day 2 when cultures became confluent, culture medium was replaced with serum-free medium. At day 3, cells were treated with 150 μM of Gap27 (SRPTEKTIFII; Biomatik, Cambridge, ON, Canada) [3,48,49], equal molar amount of Gap26 (VCYDKSFPISHVR) [3,48–50], or corresponding scrambled Gap27 (TFEPDRISITK) [33] or Gap26 (YSIVCKPHVFDRS) [50] control peptides, respectively, for up to 24 h before total RNA isolation or collection of cell lysates/conditioned medium for Western blotting. In a set of experiments, cells were treated with increasing concentrations (25, 50, 75, 100 μM) of meclofenamic acid (MFA; M4531, Sigma-Aldrich, St. Louis, MO, USA), a pharmacological connexin inhibitor [51], or corresponding amount of MFA diluent (dH_2_O) for 24 h before RNA isolation.

### Blocking C×43 Expression by siRNA Technique

To block the expression of C×43, GFBLs were seeded in 6-well plates as described above. After 24 h, siRNA transfection was carried out using Lipofectamine RNAiMax reagent (Invitrogen, Carlsbad, CA, USA). To this end, 22 μl of Lipofectamine RNAiMax and 150 picomoles of C×43 siRNAs (siRNA-1; UUUUGCAAGUGUAAACAGC or siRNA-2; AAUGAAAAGUACUGACAGC; Invitrogen) or control siRNAs (control siRNA-1 ACUUCGACACAUCGACUGC or control siRNA-2 ATCGCAAATCCGGACCTAT; Invitrogen) were mixed with 2.4 ml of Opti-MEM medium (Invitrogen), and incubated at room temperature for 5 min. The mixtures of transfection reagent and individual siRNAs were combined and incubated at room temperature for 20 min for complex formation. Then the reagent was diluted with Opti-MEM to yield final siRNA concentration of 30 nM, and added to the cells. After 5 h in cell culture incubator, the transfection reagent was removed, and cells fed with their normal growth medium overnight. Medium was then replaced with serum-free growth medium, and RNA isolation and sample collection for Western blotting was performed after 48 h.

### Real-Time PCR

Real-time PCR analysis was performed according to MIQE guidelines [52] as we have described in detail previously [53]. Briefly, total RNA from cultured GFBLs was isolated using NucleoSpin RNA II kit according to the manufacturer’s protocol (Macherey-Nagel). Total RNA (1 μg) was reverse transcribed using iScript Select cDNA Synthesis Kit (Bio-Rad, Mississauga, ON, Canada) and random oligodeoxynucleotide primers according to the manufacturer’s instructions, as described previously. The primers used for real-time PCR are listed in S2 Table. Real-time PCR amplification was performed on the CF×96 System (Bio-Rad) using the following program: 1 cycle at 94°C for 3 min 35 cycles at 94°C for 10 s, 60°C for 20 s, and reaction completion with reading plate and a melt curve analysis from 65°C to 95°C, 5 s for each 0.5°C. Amplification reactions were conducted for target genes with ubiquitin C (UBC), glyceraldehydes-3-phosphate dehydrogenase (GAPDH), hypoxanthine phosphoribosyltransferase I (Hprt1), TATAA-box binding protein (TBP), and Beta-2-microglobulin (B2M) as reference genes. For a given experiment, at least two reference genes were chosen using the integrative RefFinder tool (http://www.leonxie.com/referencegene.php). Non-transcribed RNA samples were used as a negative control. The PCR reactions were performed in triplicate for each sample. The data was analyzed and is presented based on the comparative Ct method (CFX Manager Software Version 2.1, Bio-Rad).

### Preparation of Cell Lysates/Conditioned Medium Samples for Western Blotting

Confluent GFBL cultures were treated as described above. The conditioned medium was then collected and immediately treated with Complete Protease Inhibitor Cocktail (Roche Diagnostics, Manheim, Germany). The samples were concentrated (30–40 times) by centrifugation (5,000g) using Centrifugal Filter Units (Amicon Ultra-4 3K, 3000 MWCO; Millipore, Bedford, MA, USA) for 3 h, and stored at −80°C until use. To collect cell lysates, cells were washed with ice-cold phosphate-buffered saline (PBS), and lysed with a buffer containing 25 mM Tris-HCL (pH 7.6), 100 mM Octyl β̃D-glucopyranoside, 5 mM NaF, 1 mM Na3VO4, (Sigma-Aldrich, St. Louis, MO, USA), and the Complete Protease Inhibitor Cocktail (Roche Diagnostics), dissolved in H_2_O. Lysates were collected using a rubber policeman, and filtered through a NucleoSpin Filter (Macherey-Nagel) by centrifugation at 5,000g for 10 min.

### Western Blotting

Immunoblotting analysis was conducted as described in detail previously [53]. Briefly, total protein concentration in call lysates/conditioned medium samples was determined using the Bio-Rad Protein Assay Dye Reagent Concentrate (Bio-Rad). Equal amount of protein of each sample was solubilized in SDS sample buffer containing 2-mercaptoethanol (5%) and separated by 10–12% SDS-polyacrylamide gel electrophoresis. The proteins were transferred onto a nitrocellulose membrane (Hybond-ECL membrane, GE Healthcare Bioscience, Buckinghamshire, UK). The nonspecific binding sites were blocked by incubating the membranes in Odyssey Blocking Buffer (LI-COR Biosciences; Lincoln, NE, USA) at room temperature for 1 h, followed by incubation with the primary antibody (S3 Table) at 4°C overnight. After washing with TBS containing 0.1% Tween-20 (TBS-T), the membranes were incubated with an appropriate IRdye-conjugated secondary antibody (1:10,000; LI-COR Biosciences). Dried membranes were then detected using the LI-COR Odyssey infrared reader (LI-COR Bioscience, Nebraska, USA). Intensity of the protein bands was quantitated using ImageJ software (NIH).

The activation of signaling pathways by Gap27 treatment was studied in cell lysates obtained as described above. For the experiments, GFBLs were seeded on 6-well plates, treated with Gap27 (150 μM) or equal amount of control peptide for 1, 2, 6, and 24 h, and cell lysates collected, as above. Western blotting was performed with antibodies against total or phosphorylated forms of β-Catenin and GSK3α/β (β-Catenin pathway), SMAD3 (TGF-β pathway), ERK1/2 and p38 (MAPK pathway) (S3 Table). β-Tubulin was used as a loading control.

To identify latent and active MMPs, a set of cell/conditioned medium samples was treated with or without p-aminophenylmercury acetate (APMA; 1.0 mM, pH = 7.4; Sigma-Aldrich) at 37°C for 4 h to activate latent enzymes [54] prior to gel electrophoresis and Western blotting (data not shown).

### Use of Chemical Inhibitors to Block Signaling Pathways

To determine the role of key signaling pathways in Gap27-induced gene expression, we blocked TGF-β pathway with SB431542 (20 μM; Selleckchem, Houston, TX, USA), MEK1/2 with PD184352 (10 μM; Sigma-Aldrich), p38 with SB203580 (10 μM; Cell Signaling, Danvers, MA, USA), GSK3α/β with SB415286 (30 μM; Biomol, Hamburg, Germany), AP1 with curcumin (30 μM; Sigma-Aldrich), and SP1 with WP631 (5 nM; Sigma-Aldrich) in Gap27-treated cells, respectively. To this end, confluent GFBL cultures were pre-incubated with inhibitors at 37°C for 1 h, and then treated with Gap27 (150 μM) with or without the inhibitors in serum-free growth medium for 24 h. All inhibitors were dissolved in DMSO, and control samples were treated with respective amounts of DMSO only. Total RNA was collected for real-time PCR as described above.

### Dye Transfer Experiments

To assess the GJ function of C×43, dye transfer assays were performed. To this end, GFBL cultures were generated on gelatin-coated glass coverslips in 24-well plates as described previously [55]. Briefly, the coverslips were incubated in 0.2% gelatin in PBS at 37°C for 1 h. After rinsing with PBS, coverslips were incubated in 1% glutaraldehyde at room temperature for 30 min, then washed with PBS, followed by incubation with DMEM at 37°C for 30 min. Coverslips were then washed with PBS and stored at 4°C or used immediately. To assess dye transfer through GJs by scrape loading, cells (GFBL-DC) were seeded on the coverslips in their normal growth medium as described above for 24 h, and then serum-starved in DMEM for another 24 h. Confluent cultures were then pre-incubated with Gap27 or the control peptide (150 μM; 24 h), or with MFA (50 μM; 1 h) or vehicle control (dH_2_O; 1 h) in DMEM at 37°C, media was removed, and a scrape wound was created through the cell layer with a 10 μL pipette tip, and cells incubated as above with 0.5% Lucifer Yellow (Molecular Probes Inc., Eugene, OR, USA) in PBS+ (containing 1 mM Ca^2+^ and Mg^2+^) for 5 min at 37°C. Cells were rinsed once with PBS+ and then fixed with 4% formaldehyde at room temperature for 20 min. Nuclei were then stained with 300 nM DAPI (Molecular Probes Inc.) in PBS for 5 min. In a set of experiments, cells were transfected with C×43 siRNA-1, siRNA-2 or control siRNA (30 nM) as above before seeding on the coverslips for 24 h, serum-starved for 24 h, and then subjected to the dye transfer experiment as above. Samples were mounted with Immu-Mount solution (Thermo Scientific, Pittsburgh, PA, USA), examined using the ECLIPSE 80i Microscope (Nikon, Tokyo, Japan), and images captured using NIS-Elements BR software (Version 4.20, Nikon).

### Scratch Wound Cell Migration Assay

To assess role of C×43 in cell migration, GFBLs were grown on gelatin-coated glass coverslips, serum-starved, and pretreated with Gap27 or control peptides (150 μM) for 24 h as above. A wound was then created through the cell layer using a 100 μL pipette tip. Cells were then cultured in DMEM with the peptides in a cell culture incubator, and wound closure recorded over time by standardized digital images taken from the samples using a phase contrast microscope (Nikon Eclipse, TS100) and a digital camera (Nikon Coolpix 995). In a set of experiments, cells were transfected with C×43 siRNA-1 or -2 and control siRNA-1 or -2 (30 nM) separately as above, seeded on gelatin-coated glass coverslips for 24 h, and serum-starved for 6 h before wounding. Experiments were performed in triplicate per treatment group, and images were captured from three to four different areas of the wound on each coverslip. Wound closure rate was determined measuring the area of the open wound at each time point relative to the area of the wound at the time of wounding using the Adobe Photoshop for Mac software (https://www.adobe.com).

### Immunostaining

For immunostaining, confluent GFBLs were seeded on gelatin-coated glass coverslips in their normal growth medium (24 h) and then serum starved (24 h) as described above, fixed with 4% formaldehyde at room temperature for 20 min, and then permeabilized using 0.5% Triton X-100 in PBS for 4 min. All samples were then blocked with PBS+ containing BSA (10 mg/ml) and glycine (1 mg/ml) at room temperature for 30 min, followed by an incubation with the primary antibody (S3 Table) diluted in PBS containing BSA (1 mg/ml) in a humidified chamber at 4°C overnight. The samples were then washed with PBS containing BSA (1 mg/ml) and 0.01% Triton X-100, and incubated with an appropriate Alexa-conjugated secondary antibody (1:200 dilution; Alexa 488/594; Molecular Probes Inc.) at room temperature for 1 h. Nuclei were then stained with DAPI and samples mounted as above.

For immunostaining of human gingival tissue samples, sections were fixed with cold acetone (−20°C) at room temperature for 5 min. Samples were then washed, blocked, and incubated separately with each primary antibody (S3 Table) overnight, followed by washing, incubation with appropriate Alexa 488 and 594-conjugated secondary antibodies, nuclear staining with DAPI and mounting as above. Images were acquired using optical sectioning at 1 μm (ECLIPSE 80i Microscope; Nikon), and are presented as z-stacks created by the NIS-Elements BR software (Nikon). Control stainings were performed by omitting the primary antibody incubation step.

### Statistical Analysis

All data is presented as mean +/− standard error of the mean (SEM) from repeated experiments. Statistical analysis was performed by using Student’s t-test, p<0.05 was considered statistically significant. Values obtained from the real-time PCR by the comparative Ct-method were Log2 transformed for statistical testing [56].

## Results

### C×43 Is the Major Connexin Expressed by Gingival Fibroblasts

**Figure 1:**
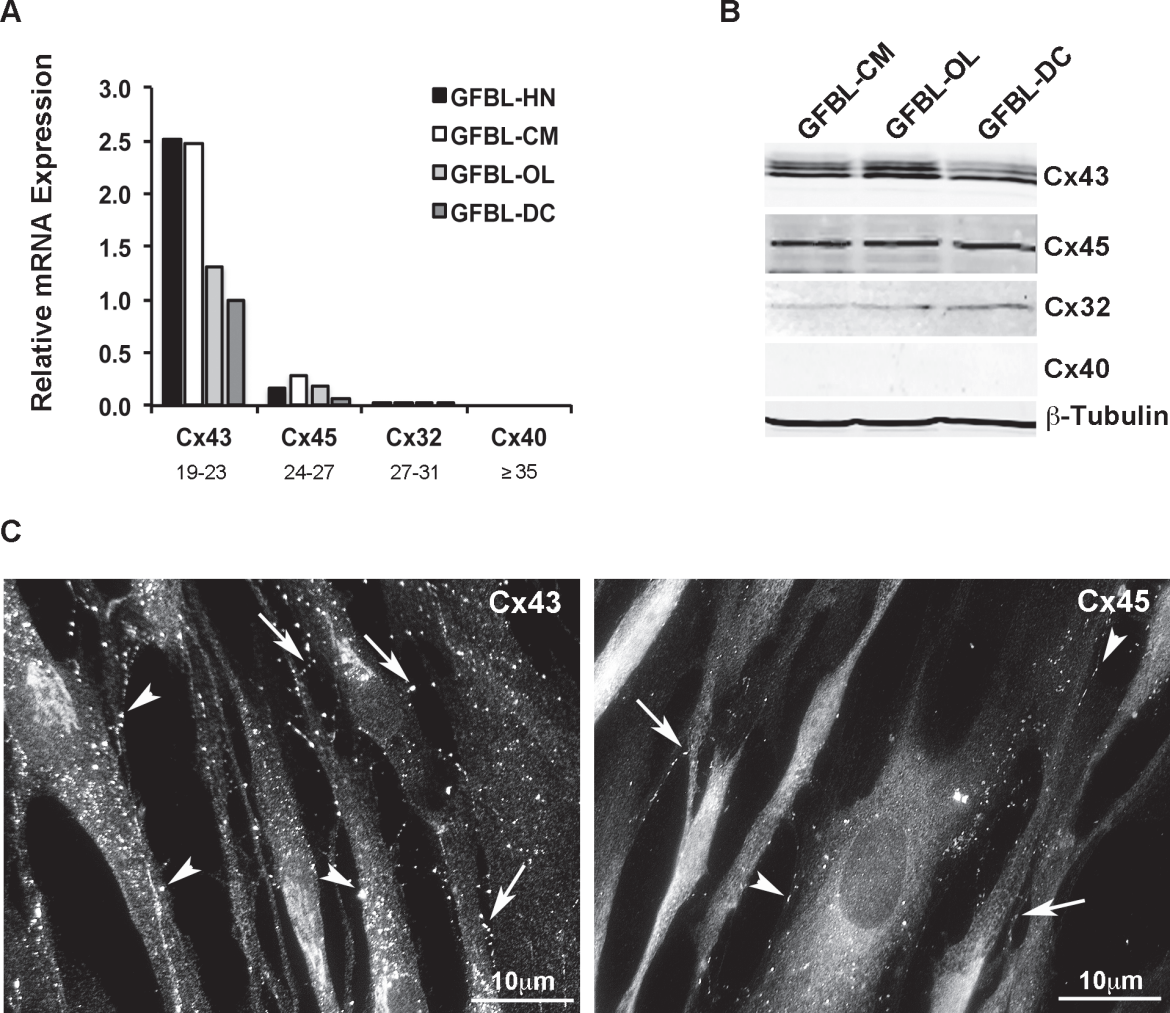
Gingival fibroblasts express C×43 as their major connexin protein. (A) Results show real-time PCR analysis of major connexins previously described in fibroblasts (C×32, C×40, C×43 and C×45) in cultured human gingival fibroblasts from four different individuals (GFBL-HN, GFBL-CM, GFBL-OL and GFBL-DC). All cell lines expressed C×43 as their major connexin, with moderate levels of C×45, low level of C×32, and no expression of C×40. Range of Ct-values obtained from real-time PCR is indicated below each gene name. (B) Similar findings were found when the same connexins were analyzed in cell lysates using Western blotting. (C) Immunostaining of a representative confluent cell culture (GFBL-DC) for C×43 and C×45. Gingival fibroblasts contained numerous C×43-positive plaques, while much fewer similar structures positive for C×45 were noted. In general, connexin-positive plaques were localized at cell-cell contact areas, possibly representing GJs (arrows), and other areas not associated with cell-cell contacts (arrowheads).

Previous studies have shown that C×43 is present in human skin, periodontal ligament and gingival fibroblasts, but that skin and periodontal ligament fibroblasts also express C×32, C×40 and C×45 [7,17,57]. Therefore, we assessed the expression of C×32, C×40, C×43 and C×45 in four parallel human gingival fibroblast (GFBL) lines from different donors in confluent monolayer cultures that allowed abundant cell-cell contacts to form. Real-time PCR (Fig. 1A) and Western blotting (Fig. 1B) analysis showed that GFBLs expressed C×43 as their major connexin protein, with moderate levels of C×45, very low levels of C×32, and no expression of C×40. Immunolocalization of C×43 and C×45 showed a punctate staining, likely representing connexin plaques. Some of these connexin plaques localized to long cellular processes contacting nearby cells likely representing GJs (Fig. 1C). In addition, they localized to areas with no apparent cell-cell contacts, possibly representing intracellular and/or cell surface non-junctional hemichannel plaques (Fig. 1C). In general, the number of C×43- compared to C×45-postive plaques was markedly higher (Fig. 1C), reflecting the real-time PCR and Western blotting analysis.

### C×43 Is Strongly Down Regulated in Gingival Epithelium and Fibroblasts During Wound Healing

**Figure 2:**
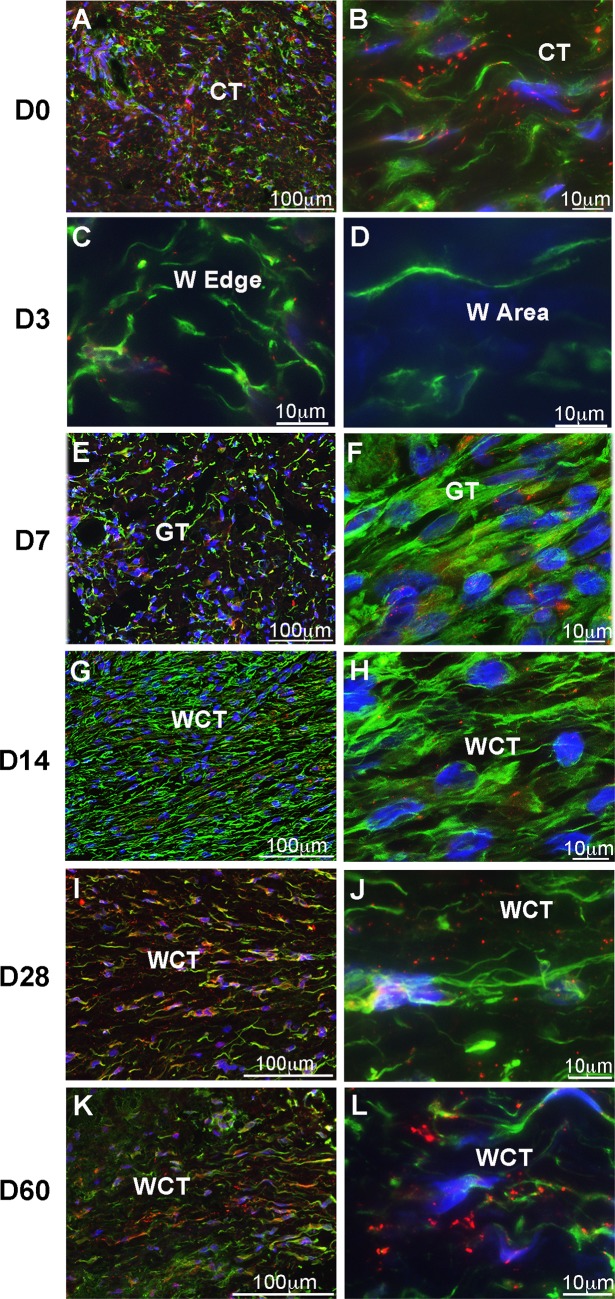
C×43 is down regulated in gingival fibroblasts during wound healing. Representative immunostainings of C×43 (red) and vimentin (green; a mesenchymal cell marker) in unwounded human oral mucosal connective tissue (attached gingiva) (A and B), and in gingival granulation and wound connective tissue 3 (C and D), 7 (E and F), 14 (G and H), 28 (I and J) and 60 days (K and L) post-wounding. (A and B) In the unwounded gingival connective tissue, abundant C×43 immunoreactivity was present as punctate staining, likely representing C×43 plaques, in vimentin-positive fibroblast-like cells throughout the tissue. (C and D) At day 3 post-wounding, C×43 was down regulated in fibroblasts at wound edge as compared to unwounded tissue (A and B). First fibroblasts that had migrated into the wound area showed no immunoreactivity for C×43 (D). (E–H) At day 7 (E and F) and 14 (G and H) post-wounding, very few C×43 positive structures were noted in fibroblasts in the highly cellular granulation and connective tissue. (I and J) At day 28 after wounding, abundance of C×43-positive plaques in connective tissue cells in the newly formed connective tissue at the wound area was increased as compared to earlier time points. However, size of these plaques was clearly smaller than in the unwounded tissue (A and B). (K and L) At day 60 after wounding, structure of the connective tissue formed at the wound area was closely similar to unwounded tissue. Size and number of C×43-positive plaques in fibroblast-like cells in the regenerated wound area was similar to the unwounded tissue (A and B). Data shown represents minimum of three sections stained in parallel samples from two to three individual donors at each time point. CT: connective tissue; W Edge: wound edge; W Area: wound area; GT: granulation tissue; WCT: wound connective tissue. Nuclear staining (blue) was performed using DAPI.

In order to assess expression of C×43 during gingival wound healing, we compared localization of C×43 in unwounded human gingiva, and in experimental excisional gingival wounds 3, 7, 14, 28 and 60 days after wounding by immunostaining (Fig. 2 and S1 Fig.). Fibroblasts were identified based on their elongated, spindle-shaped morphology, and positive immunostaining for vimentin, a mesenchymal cell marker highly expressed in fibroblasts [58]. C×43 was present in unwounded gingival epithelium where it showed strong, punctate staining in the cell-cell contacts at the spinous layer. In addition, a weaker and sparse staining was noted between some basal and granular layer cells (S1 Fig.). A punctate, positive staining for C×43, likely representing C×43 plaques [23], was also noted throughout connective tissue (Fig. 2A and B), where it associated with vimentin-positive fibroblast-like cells. Many of the C×43-positive plaques were fairly large in size (>1 μm in diameter), and some localized to long processes extending from these cells (Fig. 2B). At day 3 after wounding, abundance of C×43 was strongly reduced in the migrating wound epithelium (S1 Fig.), and in the fibroblasts residing at the wound edge and migrating into the wound (Fig. 2C and D and S1 Fig.). At day 7 when wound epithelium had completely covered the wound, and granulation tissue formation was underway, C×43 was abundantly present in 2–3 most basal cell layers of the wound epithelium, while the spinous layer showed some weak positive staining (S1 Fig.). In general, in the connective tissue cells at the wound edge (Fig. 2E and S1 Fig.), and in the wound granulation tissue (Fig. 2F and S1 Fig.), only very few C×43-positive plaques were noted. At day 14, C×43 was still most abundant in 2–3 most basal wound epithelial cell layers, but its staining was now increased also in more suprabasal cells (S1 Fig.). Our previous analysis of these same wounds has shown that at this stage, the granulation tissue had started contraction, contained abundantly α-SMA-positive fibroblasts, and was being remodeled to wound connective tissue [35,44,45]. At this stage, vimentin-stained fibroblast-like cells at the wound edge showed some C×43-positive plaques (S1 Fig.). In general, the highly cellular wound connective tissue showed only very few such structures in vimentin-positive cells (Fig. 2G and H), or in M2 macrophages (S2 Fig.), also abundantly present in these same wounds at this stage [37], or in α-SMA-positive myofibroblasts (S3 Fig.). At day 28, while the typical gingival epithelial long rete pegs were not yet formed, abundance and localization of C×43 in the epithelium at the wound site was almost similar to normal unwounded tissue, being weakly present in the basal cells, and most strongly stained in the spinous layer (S1 Fig.). At this stage, wound contraction was still underway, but cellularity and the number of myofibroblasts and M2 macrophages had dramatically decreased [35,37,44,45]. Vimentin-stained fibroblast-like cells at the wound edge and within the newly formed wound connective tissue displayed increased number of C×43-positive plaques as compared with earlier time points, but the number and size of the plaques was clearly reduced as compared to unwounded tissue (Fig. 2I and J and S1 Fig.). Few M2 macrophages that were still present in the wounds showed very little C×43 positive staining (S2 Fig.). At day 60 when the epithelium had reformed long rete pegs and connective tissue structure was normalized, the localization and abundance of C×43 was similar to unwounded tissue both at the epithelium and connective tissue at the wound site (Fig. 2K and L and S1 Fig.).

### Blocking of C×43 Function Regulates Significantly Expression of a Distinct Set of Wound Healing-Associated Genes in Gingival Fibroblasts

**Figure 3:**
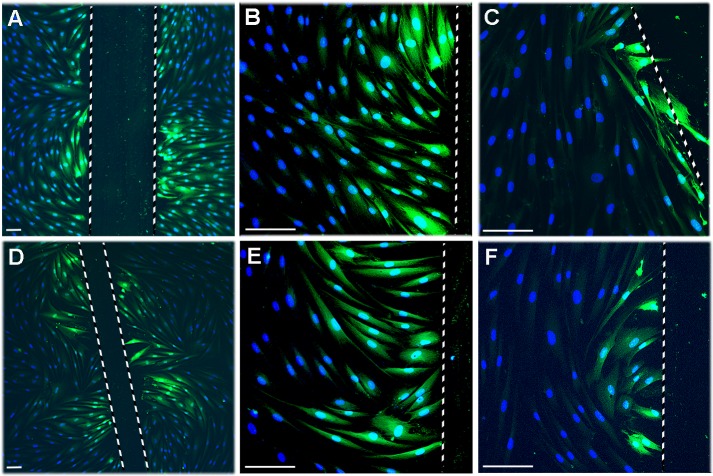
Gap27 and MFA suppress GJ-mediated dye transfer in gingival fibroblasts. (A–F) Confluent GFBL-DC cultures maintained in DMEM were scrape-loaded with Lucifer Yellow (0.5%; green) in the presence of control peptide (A and B; 150 μM), Gap27 (C; 150 μM), vehicle (dH_2_O; D and E), or MFA (50 μM; F), and dye transfer was followed for 5 min. Treatment of cells with Gap27 (C) or MFA (F) markedly reduced dye transfer as compared to control samples treated with the control peptide (A and B) or vehicle (D and E). Results show representative images from minimum of three repeated experiments. For the experiments, cells were pretreated with Gap27 and control peptide or MFA and vehicle for 24 h or 1 h before the experiments, respectively. Magnification bars: 50 μm.

Having established that C×43 is the major connexin expressed by GFBLs, and that abundance of C×43 plaques is strongly reduced during gingival wound healing, we wanted to find out the functional significance of this down regulation. Down regulation of the number of C×43-positive plaques in wound fibroblasts can result in reduced hemichannel and GJ function. Treatment of cells with C×43 mimetic peptides can be used to specifically block these channel functions [59], which promotes connective tissue wound healing *in vivo* [60]. Therefore, we treated GFBLs with Gap27, a mimetic peptide corresponding to the 11-amino acid sequence in the second C×43 extracellular loop, that blocks C×43 channel functions [59,61]. Treatment with Gap27 (150 μM) did not markedly affect cell morphology (S4 Fig.). However, it reduced GJ- mediated dye transfer, similar to meclofenamic acid (MFA), a pharmacological connexin inhibitor [51], also in this model as expected (Fig. 3). Gap27 treatment also significantly promoted GFBL migration in the scratch wound healing model (S5 Fig.), as previously described for skin fibroblasts [2]. Thus, reduced C×43 abundance/function may promote fibroblast recruitment at the early stages of human gingival wound healing. In addition to being absent from migrating fibroblasts in early wounds, C×43 abundance was also strongly suppressed in fibroblasts abundantly present inside gingival granulation and wound connective tissue at day 7–28 post-wounding. This coincides with resolution of inflammation, angiogenesis, ECM deposition, myofibroblast differentiation, contraction and remodeling stages of wound healing [37]. Therefore, we next assessed the effect of suppressing C×43 function by Gap27 in confluent cell cultures on expression of genes important specifically for these stages of wound healing. Real-time PCR results showed that Gap27 treatment significantly upregulated expression of 15, and down regulated 7, of the 54 genes analyzed (Tables 1–4). The selected genes encoded proteases (MMPs and their inhibitors), important in regulation of inflammation, angiogenesis and ECM remodeling [62,63], molecules involved in intracellular ECM degradation (Endo180 and Cathepsin K) (Table 1), ECM proteins (fibrillar and matricellular proteins, and small leucine-rich proteoglycans), cell contractility and myofibroblast-associated proteins (Table 2), TGF-β signaling associated molecules and cytokines involved in inflammation, angiogenesis and re-epithelialization (CXCL12, FGF-2, VEGF-A, IL1β, IL10 and TNF-α) [37] (Table 3), and cell-cell junction proteins (connexins and cadherins) [64] (Table 4). To rule out significant but potentially biologically irrelevant minor changes, we used minimum of +/− 1.5-fold change threshold for the significantly regulated genes. This yielded 17 significantly regulated genes of which 11 were upregulated (MMP-1, -3, -10 and -14, TIMP-1 and -3, Tenascin-C, TGF-β1, VEGF-A, C×43 and Cadherin-2), and 6 down regulated (Collagen type I, Decorin, Fibromodulin, α-SMA, NMMIIB and CXCL12) (Tables 1–4). The responses were concentration-dependent at least up to 300 μM peptide concentration (data not shown).

**Table 1:** Blocking of C×43 function with Gap27 treatment modulates significantly expression of genes involved in protein degradation during wound healing in gingival fibroblasts.

**Target Gene**	**C_t_ Value**	**Relative Expression Mean ± SEM**	**p-Value**
**MMPs and TIMPs**			
**MMP-3**	22≤Ct≤23	**6.22 ± 0.93**	***1.72608E-06
**MMP-1**	17≤Ct≤21	**4.83 ± 0.59**	***1.40031E-09
**MMP-10**	20≤Ct≤23	**4.72 ± 0.49**	***1.89712E-10
**TIMP-3**	17≤Ct≤24	**2.05 ± 0.34**	**0.001
**MMP-14**	20≤Ct≤23	**1.65 ± 0.11**	***7.93526E-05
**TIMP-1**	15≤Ct≤20	**1.63 ± 0.18**	**0.001
MMP-11	24≤Ct≤26	1.19 ± 0.07	0.16
TIMP-2	15≤Ct≤22	1.05 ± 0.05	0.30
MMP-2	13≤Ct≤16	0.98 ± 0.04	0.67
MMP-19	23≤Ct≤26	0.81 ± 0.09	0.11
TIMP-4	18≤Ct≤27	0.70 ± 0.05	***0.0001
MMP-7	Ct>30		
MMP-9	Ct>30		
MMP-12	Ct>30		
MMP-13	Ct>30		
**Molecules involved in intracellular ECM degradation**			
CTSK	17≤Ct≤19	0.88 ± 0.03	0.07
Endo180 (CD280)	19≤Ct≤21	0.83 ± 0.06	0.12

Results show real-time PCR analysis of relative mRNA expression in confluent GFBL-DC cultures treated with Gap27 (150 μM) relative to control peptide-treated samples for 24 h. Results represent mean +/− SEM from minimum of three repeated experiments (**p<0.01, ***p<0.001; Student’s t-test). Genes that are bolded show ≥1.5-fold up or down regulation relative to control peptide treated samples. Genes with negligible expression (Ct>30) were not analyzed further. MMP: Matrix Metalloproteinase; TIMP: Tissue Inhibitor of Metalloproteinase; CTSK: Cathepsin K.

**Table 2:** Blocking of C×43 function with Gap27 treatment modulates significantly expression of extracellular matrix proteins and cell contractility and myofibroblast-associated genes in gingival fibroblasts.

**Target Gene**	**C_t_ Value**	**Relative Expression Mean** **±SEM**	**p-Value**
**Fibrillar ECM proteins**			
EDA-FN	18≤Ct≤22	1.20 ± 0.09	*0.04
EDB-FN	19≤Ct≤24	1.20 ± 0.09	*0.04
Collagen type III	21≤Ct≤24	0.72 ± 0.11	0.07
**Collagen type I**	18≤Ct≤21	**0.62 ± 0.06**	***0.0007
**Matricellular proteins**			
**TN-C**	22≤Ct≤24	**5. ± 0.79**	***4.88554E-08
CTGF (CCN2)	19≤Ct≤23	1.0 ± 0.14	0.81
**Small leucine-rich proteoglygans**			
LUM	19≤Ct≤20	1.23 ± 0.13	0.09
BGN	19≤Ct≤26	1.23 ± 0.17	0.19
**DCN**	15≤Ct≤16	**0.63 ± 0.03**	***5.62708E-06
**FMOD**	24≤Ct≤27	**0.61 ± 0.06**	***0.0006
**Contractility and myofibroblast-associated genes**			
α11 integrin	26≤Ct≤27	1.27 ± 0.07	0.05
NMMIIA	21≤Ct≤22	1.20 ± 0.12	0.12
P311	26≤Ct≤27	0.89 ± 0.05	0.09
**α-SMA**	19≤Ct≤21	**0.60** **±** **0.04**	***6.79753E-06
**NMMIIB**	26≤Ct≤27	**0.59 ± 0.14**	***0.0002

Results show real-time PCR analysis of relative mRNA expression in confluent GFBL-DC cultures treated with Gap27 (150 μM) relative to control peptide-treated samples for 24 h. Results represent mean +/− SEM from minimum of three repeated experiments (*p<0.05, ***p<0.001; Student’s t-test). Genes that are bolded show ≥1.5-fold up or down regulation relative to control peptide treated samples. EDA-FN: Extra Domain A-Fibronectin; EDB-FN: Extra Domain B-Fibronectin; TN-C: Tenascin-C; BGN: Biglycan; DCN: Decorin; FMOD: Fibromodulin; LUM: Lumican; α-SMA: α-Smooth Muscle Actin; NMMIIA: Non-Muscle Myosin IIA; NMMIIB: Non-Muscle Myosin IIB.

**Table 3:** Blocking of C×43 function with Gap27 treatment modulates significantly expression of genes involved in TGF-β signaling and encoding VEGF-A and CXCL12/SDF-1α in gingival fibroblasts.

**Target Gene**	**C_t_ Value**	**Relative Expression Mean ±SEM**	**p-Value**
**TGF-β signaling related genes**			
**TGF-β1**	18≤Ct≤21	**1.60** **±0.10**	***4.64896E-06
EGR1	22≤Ct≤25	1.54 ± 0.80	0.49
TGF-β3	25≤Ct≤28	1.42 ± 0.14	*0.01
NAB1	24≤Ct≤26	1.26 ± 0.02	**0.005
NAB2	23≤Ct≤24	1.15 ± 0.26	0.59
TGF- βR2	21≤Ct≤23	0.89 ± 0.05	0.16
TGF-βR1	25≤Ct≤29	0.87 ± 0.12	0.41
TGF-β2	24≤Ct≤26	0.63 ± 0.22	0.27
EGR2	25≤Ct≤28	0.56 ± 0.13	0.12
EGR3	Ct>30		
**Growth factors and cytokines**			
**VEGF-A**	23≤Ct≤24	**3.48** **±** **0.43**	***6.29123E-07
FGF-2	23≤Ct≤24	1.19 ± 0.13	0.15
**CXCL12/SDF-1α**	20≤Ct≤21	**0.34** **±0.05**	***1.39169E-05
IL1β	Ct>30		
IL10	Ct>30		
TNF-α	Ct>30		

Results show real-time PCR analysis of relative mRNA expression in confluent GFBL-DC cultures treated with Gap27 (150 μM) relative to control peptide-treated samples for 24 h. Results represent mean +/− SEM from minimum of three repeated experiments (*p<0.05, **p<0.01, ***p<0.001; Student’s t-test). Genes that are bolded show ≥1.5-fold up or down regulation relative to control peptide treated samples. Genes with negligible expression (Ct>30) were not analyzed further. TGF-βR1: TGF-β Receptor 1; TGF-βR2: TGF-β Receptor 2; EGR1: Early Growth Response 1; EGR2: Early Growth Response 2; EGR3: Early Growth Response 3; NAB1: NGFI-A Binding Protein-1; NAB2: NGFI-A Binding Protein-2; VEGF-A: Vascular Endothelial Growth Factor-A; FGF-2: Fibroblast Growth Factor-2; IL1β Interleukin-1β; IL Interleukin-10; TNF-α: Tumor Necrosis Factor-α.

**Table 4:** Blocking of C×43 function with Gap27 treatment upregulates significantly expression of C×43 and Cadherin-2 expression involved in formation of cell-cell junctions.

**Target Gene**	**C_t_ Value**	**Relative Expression Mean ±SEM**	**p-Value**
**Cell-cell junction molecules**			
**C×43**	21≤Ct≤23	**1.79 ± 0.15**	***3.05801E-05
**Cadherin-2**	22≤Ct≤23	**1.68 ± 0.21**	**0.002
C×45	24≤Ct≤27	1.18 ± 0.15	0.21
Cadherin-11	21≤Ct≤22	1.03 ± 0.06	0.69
C×32	27≤Ct≤30	0.95 ± 0.24	0.85

Results show real-time PCR analysis of relative mRNA expression in confluent GFBL-DC cultures treated with Gap27 (150 μM) relative to control peptide-treated samples for 24 h. Results represent mean +/− SEM from minimum of three repeated experiments (*p<0.05, **p<0.01, ***p<0.001; Student’s t-test). Genes that are bolded show ≥1.5-fold up or down regulation relative to control peptide treated samples. C×43: Connexin 43; C×45: Connexin 45; C×32: Connexin 32.

**Figure 4:**
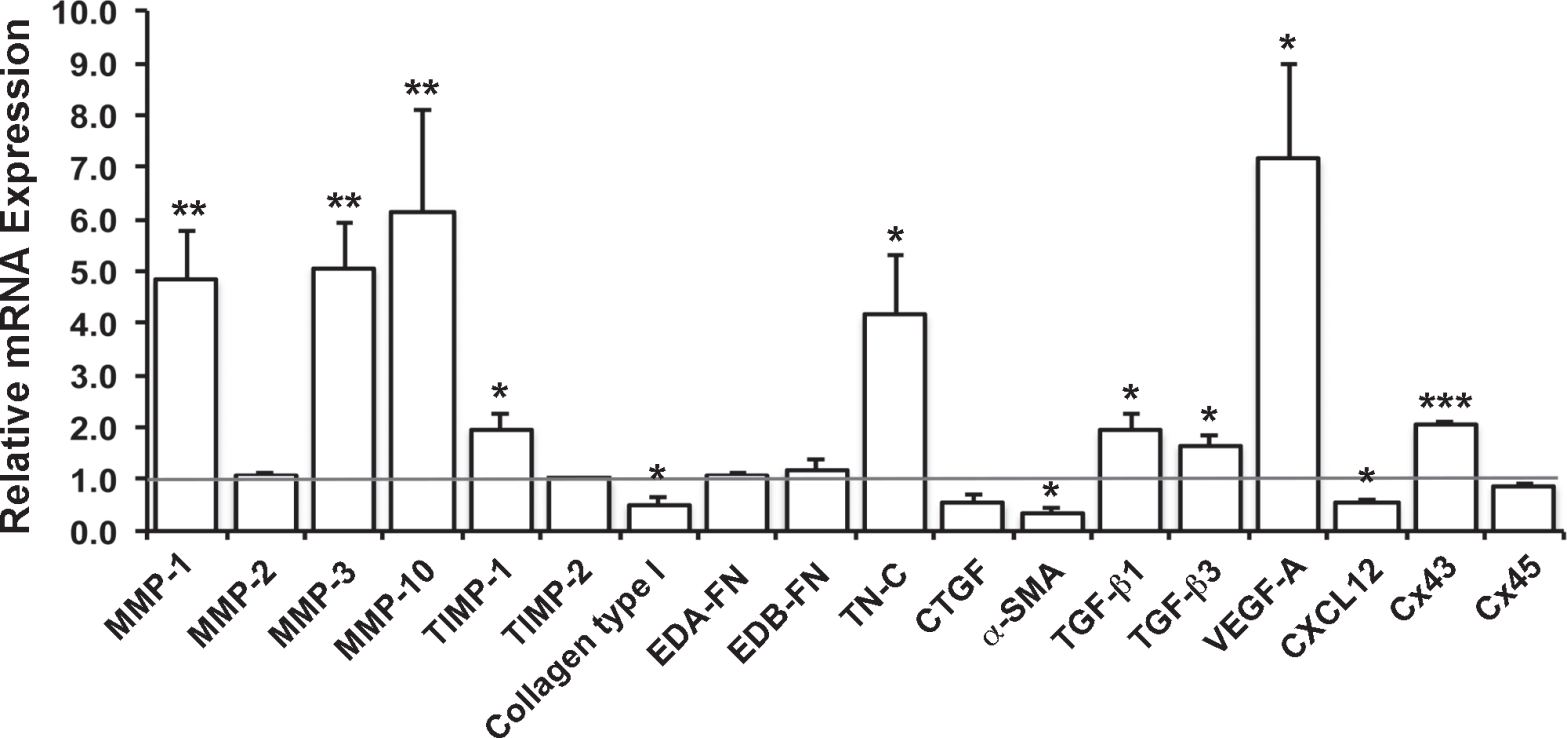
Effect of Gap27-mediated blocking of C×43 function on gene expression in parallel gingival fibroblast lines. Confluent cultures of gingival fibroblasts from three different individuals (GFBL-HN, GFBL-CM and GFBL-DC) were treated with Gap27 or control peptide (150 μM) for 24 h, and expression of a set of genes involved in wound healing was analyzed by real-time PCR. Results represent mean +/− SEM of mRNA expression relative to control peptide-treated cells from triplicate samples in one experiment (*p<0.05, **p<0.01, ***p<0.001; Student’s t-test). Horizontal line indicates relative mRNA expression for the control-peptide treated samples. EDA-FN: Extra Domain A-Fibronectin; EDB-FN: Extra Domain B-Fibronectin; TN-C: Tenascin-C; CTGF: Connective Tissue Growth factor (CCN2); α-SMA: α-Smooth Muscle Actin; VEGF-A: Vascular Endothelial Growth Factor-A.

In order to find out, whether the Gap27-mediated regulation of gene expression is a common property of GFBLs, we assessed its effect on expression on a set of the above genes in three parallel GFBL lines in the same experiment (Fig. 4). The findings confirmed a gene expression response to Gap27 treatment in all GFBL lines that was consistent with initial findings using GFBL-DC (Tables 1–4).

To confirm specificity of the Gap27 treatment, we also blocked C×43 function by Gap26, another connexin mimetic peptide corresponding to a 13 amino acid sequence in the first C×43 extracellular loop [59,61], and by MFA, and assessed expression of a set of genes as above. Gap27 and Gap26 treatments caused in general similar gene expression changes in GFBLs, although the magnitude of change slightly varied (S6 Fig.). MFA treatment induced a concentration-dependent increase in MMP-1 and -10, Tenascin-C and VEGF-A expression, and down regulated CXCL12 (S7 Fig.), similar to Gap27 (Tables 1–3) and Gap26 treatment (S6 Fig.). Interestingly, Gap27 (Table 4), Gap26 (S6 Fig.) and MFA (S7 Fig.) induced up to about 2-fold increase in expression of C×43 mRNA. Likewise, Gap27 and Gap26 caused about 2-fold increase in C×43 protein level, while MFA had no effect (data not shown).

In a set of experiments, we also suppressed C×43 expression in three parallel GFBL lines using two different C×43 siRNAs (C×43 siRNA-1 and -2), and studied the expression of the above wound healing-associated genes. Treatment of GFBLs with both C×43 siRNAs consistently resulted in about 80% down regulation of C×43 at both mRNA and protein levels, while expression of the two other connexins expressed by these cells, C×32 and C×45, were not affected (data not shown). C×43 siRNA treatment with both C×43 siRNA-1 and -2 effectively suppressed GJ-mediated dye transfer (S8 Fig.), but did not have a significant effect on fibroblast migration in the scrape-wound assay (S5 Fig.). Similar to Gap27 treated cells, C×43 siRNA treatments significantly increased expression of Tenascin-C (1.46 +/− 0.15-fold change; p<0.05) and reduced expression of Collagen type I (0.89 +/− 0.03-fold change; p<0.001) as compared to control treatment. However, possibly due to incomplete C×43 down regulation, the gene expression responses to C×43 siRNA treatment were small with none of the studied genes reaching the +/− 1.5-fold change threshold (data not shown).

### Characterization of Proteins Regulated by Gap27 Treatment in Gingival Fibroblasts

**Figure 5:**
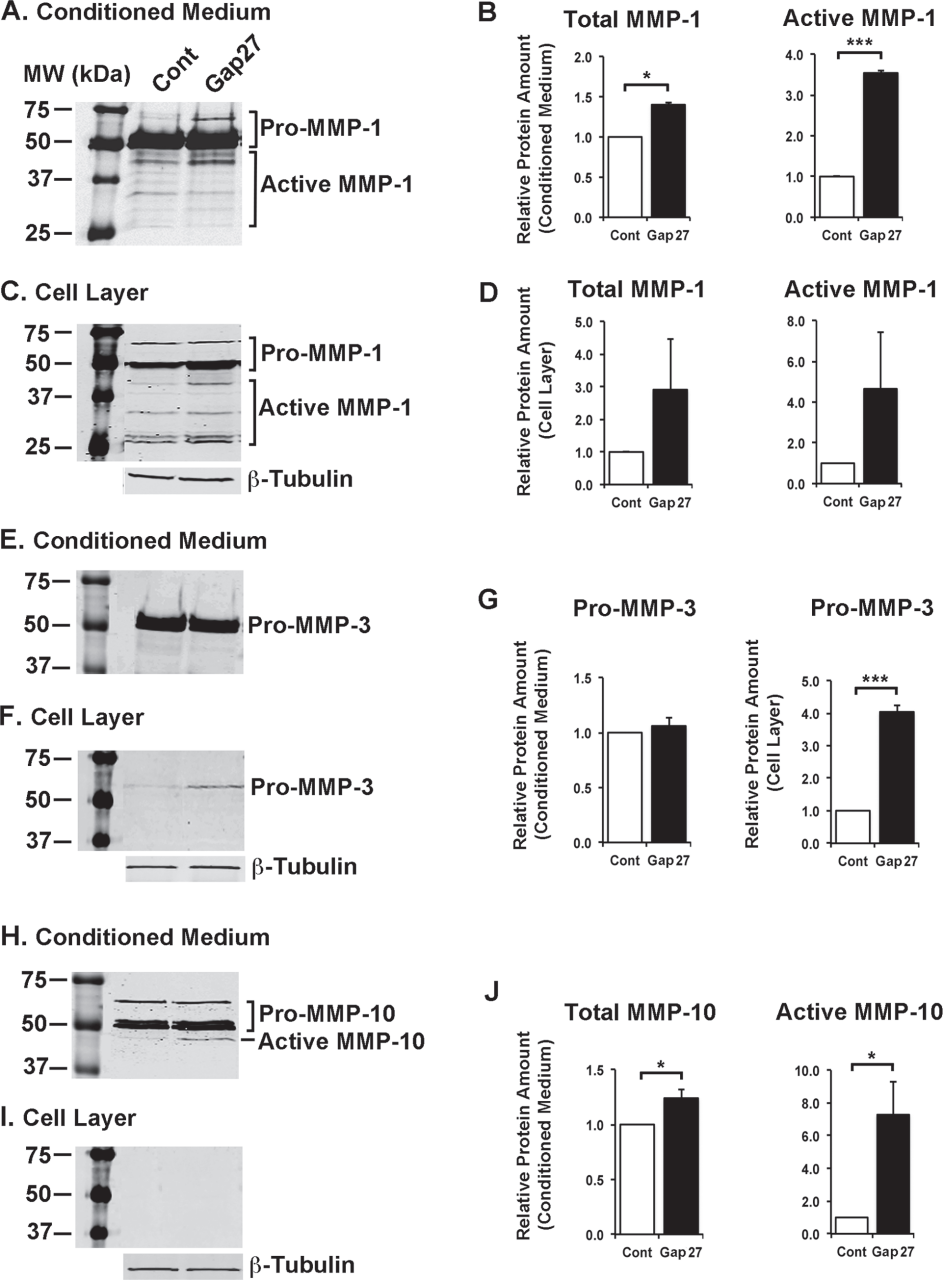
Blocking of C×43 by Gap27 resulted in significantly increased secretion of active MMP-1 and MMP-10, and pro-MMP-3 by gingival fibroblasts. Confluent cultures of gingival fibroblasts (GFBL-DC) were treated with Gap27 or control peptide (150 μM) for 24 h, and abundance of MMP-1 (A–D), MMP-3 (E–G), and MMP-10 (H–J) in the conditioned medium and cell layer was analyzed by Western blotting. (B, D, G and J) Quantitation of MMP levels in Western blots shows mean +/− SEM from three independent experiments (*p<0.05, ***p<0.001; Student’s t-test). Sample loading for cell layer fraction was normalized for β-Tubulin levels. Identity of active and pro-forms of the enzymes was confirmed by pretreatment of a set of samples with or without APMA to activate latent enzymes prior to Western blotting (data not shown).

**Figure 6:**
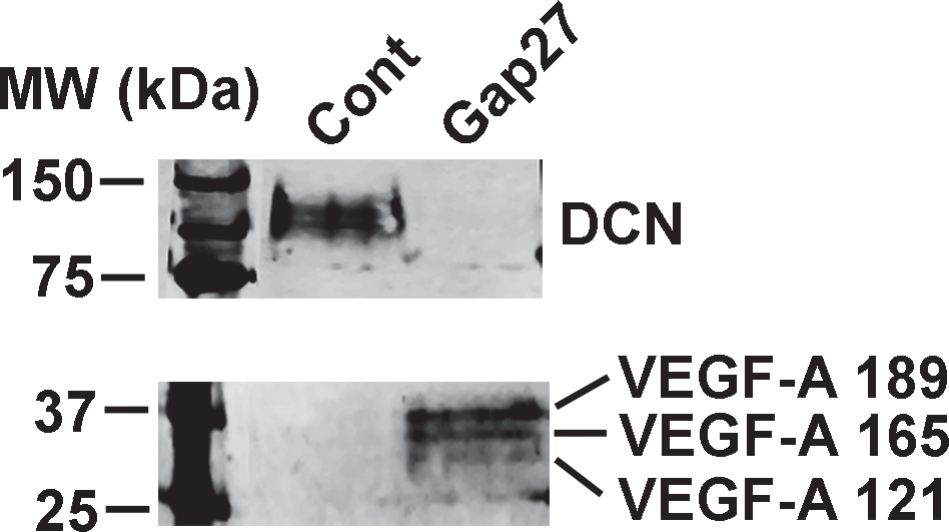
Blocking of C×43 function by Gap27 promotes secretion of VEGF-A, and suppresses DCN levels in gingival fibroblast cultures. Confluent cultures of gingival fibroblasts (GFBL-DC) were treated with Gap27 or control peptide (150 μM) for 24 h, and Vascular Endothelial Growth Factor-A (VEGF-A) and Decorin (DCN) levels were analyzed in the conditioned medium by Western Blotting. Representative results from three independent experiments are shown.

**Figure 7:**
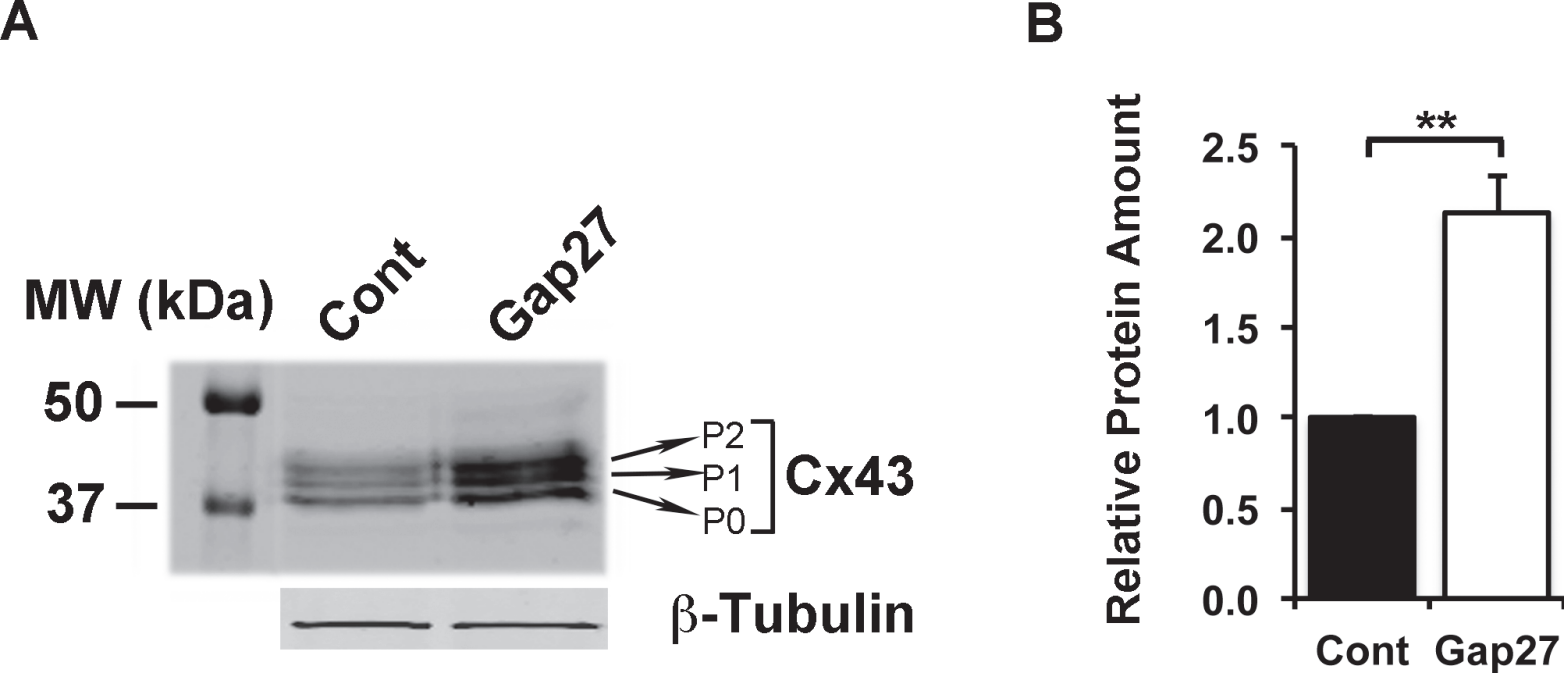
Gap27 treatment increases C×43 protein abundance significantly. (A) Confluent cultures of gingival fibroblasts (GFBL-DC) were treated with Gap27 or control peptide (150 μM) for 24 h, and abundance of C×43 was analyzed by Western blotting. Gap27 treatment did not affect the relative intensities of three bands corresponding to differently phosphorylated forms of C×43 (P0: pS368; P1:pS279/282 and pS255; P2: pS262). (B) Quantitation of C×43 levels in Western blots shows mean +/− SEM from three independent experiments (**p<0.01; Student’s t-test). Sample loading was normalized for β-Tubulin levels.

As gene expression analysis showed that blocking of C×43 function by Gap27 strongly regulated expression of several genes (Tables 1–4), we further assessed the protein levels of a set of genes that showed significant, minimum of +/− 1.5-fold change relative to control treatment in the real-time PCR analysis. Western blotting analysis showed that treatment of GFBLs with Gap27 (Fig. 5) resulted in markedly increased secretion of active and/or total MMP-1 (Fig. 5A and B) and −10 (Fig. 5H and J) compared to control treatments. In addition, Gap27-treated cells produced significantly elevated levels of pro-MMP-3 in the cell layer (Fig. 5F and G). Expression of mRNA for Decorin (Table 2), a small leucine-rich proteoglycan that regulates cell functions involved in wound healing and fibrosis, and VEGF-A (Table 3), a potent pro-angiogenic growth factor [37], were also strongly suppressed and upregulated, respectively, by Gap27 treatment. Accordingly, Western blotting showed robust down regulation of Decorin and upregulation of VEGF-A levels in the cell culture medium of Gap27-treated GFBL cultures (Fig. 6). Gap27 treatment also significantly increased C×43 expression at mRNA (Table 4) and total protein levels (Fig. 7). However, no changes in the relative intensities of the three bands corresponding to the differently phosphorylated forms of C×43 recognized by the C×43 antibody (P0: pS368; P1:pS279/282 and pS255; P2: pS262) [65,66] were noted in the Western blots (Fig. 7A).

### Blocking of C×43 Function by Gap27 Modulates Key Signaling Pathways in Gingival Fibroblasts

**Figure 8:**
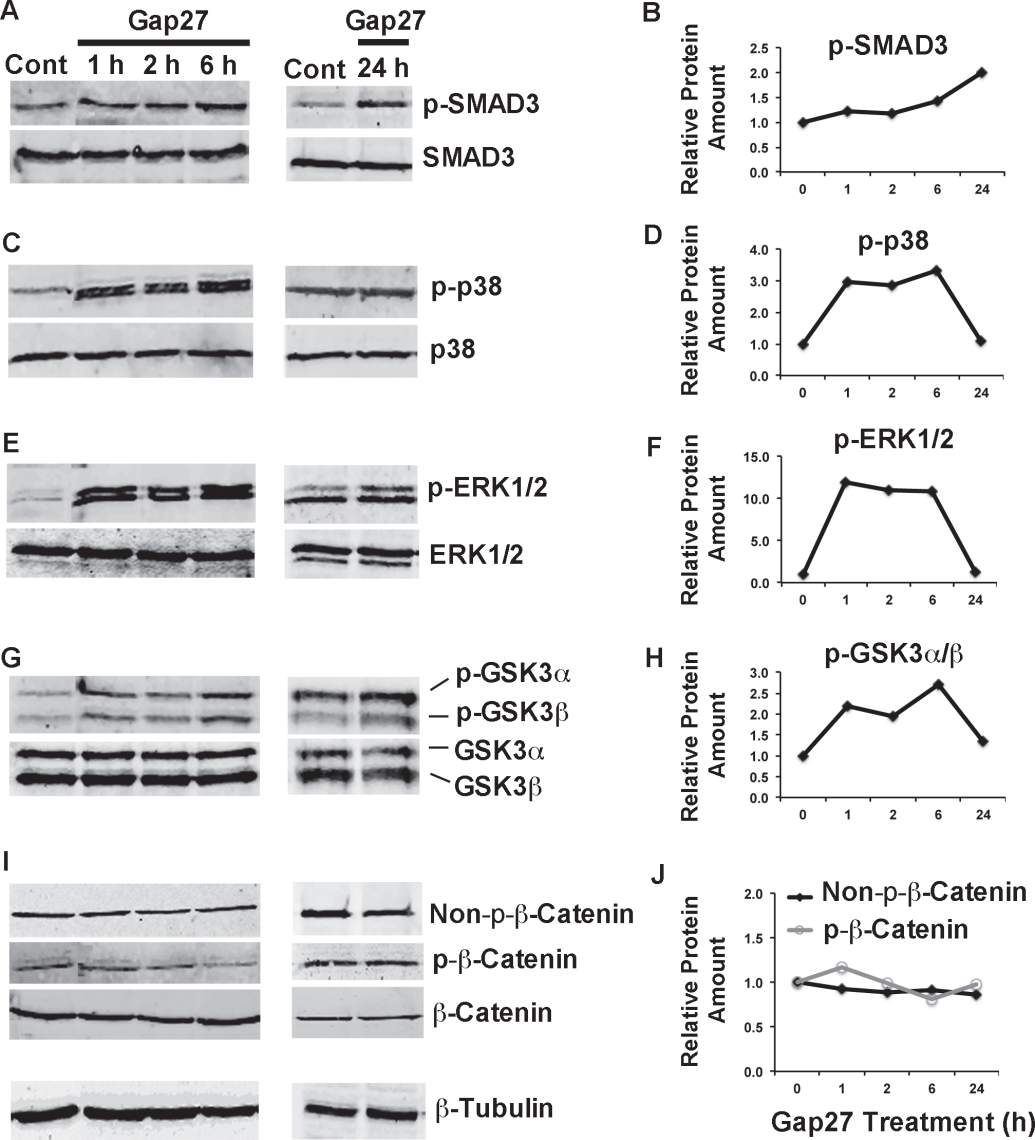
Western blotting analysis of key signaling pathways modulated by Gap27 in gingival fibroblasts. Confluent cultures of gingival fibroblasts (GFBL-DC) were treated with Gap27 or control peptide (150 μM) for 1, 2, 6, and 24 h. Cell lysates were analyzed for protein levels of total SMAD3 and phosphorylated SMAD3 (p-SMAD3) (A), total p38 and phosphorylated p38 (p-p38) (C), total ERK1/2 and phosphorylated ERK1/2 (p-ERK1/2) (E), total GSK3α/β and phosphorylated GSK3α/β (p-GSK3α/β) (G), and total β-Catenin, phosphorylated β-Catenin (p-β-Catenin) and non-p-β-Catenin (I). (B, D, F, H and J) Quantitation of the phosphorylated or non-phosphorylated signaling molecules relative to their total levels at time 0 (control samples), and at 1, 2, 6 and 24 h after Gap27 treatment. Sample loading was normalized for β-Tubulin levels. Results from one experiment are shown.

Having established that during gingival wound healing, C×43 abundance and/or GJ and hemichannel functions maybe strongly reduced, and that suppressing C×43 function in cultured fibroblasts distinctly regulates expression of a set of wound healing-associated genes, we wanted to find out which intracellular signaling pathways are involved. To this end, we treated GFBLs with Gap27 and assessed phosphorylation changes in TGF-β (SMAD3), MAPK (ERK1/2 and p38), GSK3α/β and β-Catenin pathways that have been previously associated with C×43-mediated signaling [6,67–73], and wound healing and fibrosis [74–78] (Fig. 8). Gap27 induced robust phosphorylation of p38 and ERK1/2 already after 1 h after treatment. These responses lasted at least for 6 h, before returning to the levels of untreated cells by 24 h (Fig. 8C–F). Gap27 treatment also markedly increased GSK3α/β phosphorylation at 1–6 h, returning to the normal level by 24 h (Fig. 8G and H), while phosphorylated and non-phosphorylated β-Catenin levels, downstream targets of GSK3α/β, did not show marked changes (Fig. 8I and J). Gap27 treatment did not noticeably affect SMAD3 phosphorylation until 24 h, when the steady-state p-SMAD3 levels were markedly increased (Fig. 8A and B). Thus, in gingival fibroblasts, blocking of C×43 by Gap27 treatment induced fast activation of MAPK and GSK3α/β signaling pathways, while TGF-β pathway was activated via a slower, possibly indirect, mechanism.

### Distinct Involvement of Gap27-Regulated Signaling Pathways in Modulation of Wound Healing-Associated Genes in Gingival Fibroblasts

In order to further assess the role of the above pathways in Gap27-induced gene expression changes, we blocked TGF-β signaling by SB431542, MEK1/2 by PD184352, p38 by SB203580, and GSK3α/β by SB415286 in Gap27-treated cells, and assessed gene expression changes relative to untreated cells by real-time PCR. In addition, we assessed involvement of AP1 and SP1, transcription factors previously linked to C×43 signaling [73,79,80], by treating cells with curcumin and WP631, respectively. We specifically focused on assessing expression of the genes that were significantly modulated (with a minimum by +/− 1.5-fold change threshold) by Gap27 in the above experiments. Results showed that MEK1/2 signaling was most widely involved in Gap27-induced change in gene expression, as its inhibition totally blocked Gap27-induced expression change of 10 genes. These genes included proteases and their inhibitors (MMP-1, -3 and -10, TIMP-1 and -3), ECM molecules (Collagen type I and Tenascin-C), cell contractility-associated genes (α-SMA and NMMIIB), and Cadherin-2 (Table 5 and S9 Fig.). In addition, this pathway partially regulated (inhibited Gap27-induced expression change by at least 50%) expression of growth factors (TGF-β1 and VEGF-A) and C×43. The only genes that were not affected by blocking of MEK1/2 were MMP-14, two leucine-rich proteoglycans (Decorin and Fibromodulin), and CXCL12. The other examined pathways also totally or partially regulated Gap27-modulated expression of several genes. For instance, inhibition of GSK3α/β resulted to total inhibition of Gap27-induced upregulation of TIMP-1 and -3 expression, while it partially blocked MMP-1 and TGF-β1 upregulation. Findings also showed variable and complex interplay between the studied pathways in regulation of Gap27-induced gene expression. For instance, Gap27-induced MMP-10 upregulation and Collagen type I down regulation was only blocked by the MEK1/2 inhibitor. In contrast, Gap27-induced TIMP-1 expression was totally blocked by SP1, TGF-β, MEK1/2 and GSK3α/β inhibitors, and partially by p38 inhibitor. On the other hand, Gap27-induced Tenascin-C expression was totally inhibited by AP1 and MEK1/2 inhibitors, and partially by SP1, TGF-β and p38 inhibitors. Transcription factors AP1 or SP1 were distinctly involved in regulating 7 of the Gap27-modulated genes. Interestingly, inhibition of SP1 totally blocked Gap27 induced upregulation of C×43 expression, while blocking of AP1, p38 and MEK1/2 had a partial effect. Gap27-induced upregulation of VEGF-A and down regulation of CXCL12 were only partially blocked by MEK1/2 and SP1 inhibitors, respectively, suggesting that also other unidentified pathways are involved. In addition to the above effects, interestingly, blocking of GSK3α/β strongly potentiated Gap27-induced upregulation of MMP-3 (S9 Fig.) and Tenascin-C expression (S9 Fig.). Moreover, Gap27 treatment with simultaneous inhibition of MEK1/2 resulted to strongly induced α-SMA expression (by more than 7-fold), while treatment with Gap27 alone reduced its expression by about 50% (S9 Fig.).

**Table 5:** Blocking of C×43 function with Gap27 treatment activates distinct signaling pathways that regulate wound healing-associates genes in gingival fibroblasts.

**GENE**	**AP1**	**SP1**	**TGF-β**	**p38**	**MEK1/2**	**GSK3α/β**
MMP-1					**T**	P
MMP-3		P		**T**	**T**	
MMP-10					**T**	
MMP-14			P	P		
TIMP-1		**T**	**T**	P	**T**	**T**
TIMP-3	P		P	**T**	**T**	**T**
Collagen type I					**T**	
TN-C	**T**	P	P	P	**T**	
DCN	P		**T**			
FMOD		P	P			
α-SMA	P				T	
NMMIIB			P		**T**	
TGF-β1	P		P	**T**	P	P
VEGF-A					P	
CXCL12/SDF-1α		P				
C×43	P	**T**		P	P	
Cadherin-2	**T**	**T**	P	**T**	**T**	

Results show a summary of involvement of AP1, SP1, TGF-β p MEK1/2 and GSK3α̃β signaling pathways in Gap27-mediated regulation of gene expression in GFBLs. Results were obtained from real-time PCR analysis of relative mRNA expression in confluent GFBL-DC cultures treated with Gap27 (150 μM) with or without corresponding signaling pathway inhibitors for 24 h, and show results relative to control peptide/vehicle treated samples. T: Inhibition of the pathway completely blocks Gap27-induced change in gene expression. P: Inhibition of the pathway partially (by at least 50%) blocks Gap27-induced change in gene expression. TN-C: Tenascin-C; DCN: Decorin; FMOD: Fibromodulin; α-SMA: α-Smooth Muscle Actin; NMMIIB: Non-Muscle Myosin IIB; VEGF-A: Vascular Endothelial Growth Factor-A.

## Discussion

Gingival fibroblasts that participate in scarless oral wound healing expressed C×43 as the major connexin, which is similar to skin fibroblasts [2,21]. In oral wounds, C×43 expression was strongly down regulated in gingival wound epithelium during epithelial migration stage. This is in agreement with studies assessing C×43 localization in the epithelium of murine skin and buccal mucosal, and in human skin wounds [10–15,38]. Thus far, very little was known about C×43 expression and localization in connective tissue cells during wound healing. Interestingly, in gingival fibroblasts, which were identified based on morphological criteria and positive immunostaining for the mesenchymal cell marker vimentin [58], strong C×43 immunoreactivity localized to large plaque-like structures in unwounded tissue. In order to form functional GJs, connexins cluster on the cell membrane to form plaques. Cell culture findings suggest that connexins are first transported to the cell membrane as hemichannels where they then join GJ plaques directly or by moving laterally towards them [5,81]. The size of the plaques can reach few micrometers [81], and can be therefore detected by immunostaining. In contrast, individual connexins or hemichannels are undetectable by this method [5,82]. Atomic force microscopy of cardiac GJs has also suggested existence of large (up to 2 μm^2^) hemichannel plaques [83]. Connexins can also be present intracellularly during synthesis and transport to the cell membrane, and when being endocytosed or assembled to the mitochondrial membrane. However, these intracellular connexins have not been reported to organize into large plaque-like structures [5]. Thus, the C×43-positive structures observed in gingival fibroblasts *in vivo* likely represent C×43 hemichannel and/or GJ plaques on the cell membrane.

Interestingly, the C×43-positive plaques found in fibroblasts in unwounded tissue were missing from fibroblasts at the wound edge and those migrating into the wound at days 3 and 7 post-wounding. This could be explained by reduced recruitment of C×43 to hemichannel and GJ plaques, or their redistribution, resulting to smaller plaques undetectable by immunostaining, or from down regulation of C×43 expression and/or its increased turnover. The factors that modulate these processes during wound healing *in vivo* are not clear, but are likely cell type-specific and could include effects of wound healing-related cytokines, growth factors and mechanosignaling [5,84]. In any case, similar to cultured skin fibroblasts [2], blocking of C×43 function by Gap27 promoted gingival fibroblast migration, suggesting that C×43 may regulate fibroblast recruitment into the wound provisional matrix. Interestingly, C×43 was also still largely absent from the vimentin-positive cell population established in the wound at day 14 and 28 post-wounding. These cells could include fibroblasts, myofibroblasts and macrophages that all express vimentin [85,86]. Reparative M2 macrophages are abundant and the predominant macrophages in these same gingival wounds at day 14 and 28 post-wounding [37]. However, our double immunostaining showed that very few C×43 plaques were detected in M2 macrophages at this stage. Previous studies have not explored C×43 in wound macrophages. However, contrary to our findings, M2 macrophages in thyroid tumor stroma show abundant C×43-positive plaques [87]. Our previous analysis has also shown that the number of α-SMA-rich myofibroblasts is strongly increased, and wound contraction is underway, already at day 14 in these same wounds [35,44,45]. However, only few C×43-positive plaques were noted in areas where myofibroblasts were abundant at this stage. Therefore, it is possible that in human gingival wounds absence of C×43 plaques promotes myofibroblast differentiation and wound contraction. In support of this, suppressing C×43 expression or function in murine models of wound healing results to earlier recruitment and disappearance of myofibroblasts from the wounds, and reduced wound connective tissue size [20], suggesting that in these wounds myofibroblast differentiation and contraction occurred earlier and/or was accelerated. Curiously though, in cultured rat cardiac fibroblasts, C×43 positively regulates α-SMA expression [88], and in mouse fibroblasts, C×43 deficiency associates with a reduced ability of the cells to contract a collagen gel, a model for wound contraction [89]. In addition, our findings showed that blocking of C×43 function by Gap27 significantly reduced expression of α-SMA and non-muscle myosin IIB (NMMIIB), another cytoskeletal protein involved in tissue contraction [90]. Thus, role of C×43 function in α-SMA expression, myofibroblast differentiation and contraction appears cell- and context-dependent, and requires further investigation. Nevertheless, it is possible that the early reduction of C×43 plaques, and their slow reformation in gingival fibroblasts at the late stages of wound healing has functional significance for the scarless gingival wound-healing outcome. In particular, reduced abundance of C×43 plaques at days 14 and 28 post-wounding in wound fibroblasts coincides not only with myofibroblast differentiation and contraction, but also with resolution of inflammation, angiogenesis, ECM deposition and remodeling stages of wound healing [37, 43–46], suggesting a role for C×43 in modulating also these events.

To study the significance of reduced C×43 function for these later stages of wound healing, we blocked its function in human gingival fibroblasts by C×43 mimetic peptide Gap27 [3]. Our findings showed that Gap27 reduced GJ-mediated dye transfer as expected, confirming its inhibitory effect also in human gingival fibroblasts. Furthermore, Gap27 treatment significantly regulated a number of genes that maybe beneficial for scarless gingival wound healing. These effects were not limited to Gap27, as similar results were also obtained by using Gap26, another C×43 mimetic peptide [3]. It is interesting that the mimetic peptides significantly upregulated several genes. This suggests that for those genes that were upregulated by the treatment, normal C×43 function is inhibitory, *i.e.* it is needed to suppress expression of these genes. Therefore, during gingival wound healing reduced expression/function of C×43 in fibroblasts may allow increased expression of these molecules. This group of molecules includes several MMPs, TIMP-1 and -3, Tenascin-C, TGF-β1 and VEGF-A, which are important modulators of inflammation, cell migration, angiogenesis and ECM deposition [91], and may contribute to reduced inflammation and efficient angiogenesis found in gingival wounds [36,37]. Of note, Tenascin-C accumulation is strongly induced in early granulation tissue of human gingival wounds [35,42], suggesting that down regulation of C×43 expression or function may drive this process *in vivo*. The hallmark of scar formation and fibrosis is increased accumulation of ECM and increased cell contractility [92]. Therefore, it is also interesting to note that blocking of C×43 function in gingival fibroblasts caused a robust down regulation of several ECM (Collagen type I, Decorin and Fibromodulin) and cell contractility-associated genes (α-SMA and NMMIIB). Interestingly similar to our findings, comparable treatment of human skin fibroblasts with Gap27 was also recently shown to significantly increase expression of MMP-1 mRNA [33], while unlike in gingival fibroblasts, Collagen type I and CTGF, two genes strongly associated with scar formation and fibrosis *in viv*o [92], were significantly upregulated [33]. There is increasing evidence that human skin and gingival fibroblasts are phenotypically distinct [36,37,53,93]. Therefore, it is possible that different function of C×43 in skin and gingival fibroblasts may in part contribute to the different wound healing outcomes in these two tissues, but this needs further experimental verification.

The mechanisms of the mimetic peptide-induced gene expression change in gingival fibroblasts may include blocking of transfer of signaling molecules via hemichannels and/or GJs, peptide-induced changes in C×43 levels, conformation and/or phosphorylation of its cytoplasmic tail that interacts with the signaling molecules [3,6,72,94]. The latter channel-independent effects maybe mediated via C×43 cytoplasmic tail that recruits and interacts with intracellular signaling effectors, including MAPK (ERK1/2 and p38), GSK3/β-Catenin and PI3K-Akt-GSK3 pathway mediators [6,67,69–73] also involved in wound healing and scar formation [75,76]. In addition, C×43 competes with SMAD2/3 for binding to tubulin releasing SMAD2/3 from the microtubules and promoting TGF-β signaling [68]. Our findings showed that MFA, a pharmacological connexin channel inhibitor [51], also blocked GJ-mediated dye transfer in gingival fibroblasts, and induced similar changes in the expression of a set of genes as the C×43 mimetic peptides, suggesting that the noted Gap27-induced gene expression changes depended on the channel functions of C×43. However, Gap27 also caused a robust activation of the above C×43 cytoplasmic tail-mediated signaling pathways. Thus, regulation of both channel-dependent and -independent functions maybe involved in Gap27-regulated gene expression in gingival fibroblasts.

In order to study the role of the above signaling pathways in regulation of Gap27-induced fibroblast gene expression in more detail, we used pathway-specific pharmacological inhibitors. In addition, we blocked two transcriptional regulators (AP1 and SP1) that associate with C×43 signaling [73,79,80]. Blocking of these pathways distinctly, and co-operatively regulated Gap27-modulated gene expression, and most notably, as Gap27 caused a robust early activation (phosphorylation) of ERK1/2 and p38 MAPKs, pharmacological blocking of these pathways totally or partially blocked Gap27-modulated expression of 14 out of the 17 genes analyzed. Inhibition of MEK1/2 alone, an upstream regulator of ERK1/2 [95], totally blocked Gap27-induced change of 10 genes. These included MMP-10 and Collagen type I that were not affected by the other pathways studied. Thus, MEK-ERK1/2 pathway is a major target of Gap27-induced signaling in gingival fibroblasts. Whether this depends on the reported ability of MAPKs to regulate C×43 channel functions and phosphorylation of the cytoplasmic tail [72] remains to be shown.

As mentioned above, C×43 can also promote TGF-β-induced signaling [68]. Interestingly, Gap27 treatment did not affect phosphorylation of SMAD3 during the first 6 h after treatment. However, an increased level of p-SMAD3 was noted after 24 h. Therefore, Gap27 induces activation of TGF-β pathway in gingival fibroblasts, but this may occur via a distinct, indirect mechanism. The inhibitory effect of Gap27 is time dependent, as it blocks hemichannels within minutes to few hours, while GJ inhibition may require up to 24 hours to occur [3]. Thus, the late activation of SMAD3 after Gap27 treatment may also depend on its distinct effects on GJs rather than hemichannels. In any case, pharmacological inhibition of TGF-β signaling totally or partially suppressed Gap27-induced change in expression of 9 of the studied genes, indicating that this C×43-mediated pathway has a role in modulating cell functions relevant to wound healing.

Treatment of gingival fibroblasts with Gap27 also induced fast phosphorylation of GSK3α/β, but this did not associate with marked changes in the phosphorylation or levels of its downstream target β-Catenin. Phosphorylation of GSK3α/β renders it inactive removing its inhibitory effect on its targets, which include more than 40 proteins and transcription factors [96]. Therefore, C×43-mediated phosphorylation of GSK3α/β likely affects other downstream targets than the β-Catenin pathway. Involvement of this pathway is supported by the finding that pharmacological blocking of phosphorylation of GSK3α/β totally blocked TIMP-1 and -3, and partially MMP-1 and TGF-β1 upregulation induced by Gap27 treatment. Furthermore, blocking of GSK3α/β potentiated Gap27-induced expression of MMP-3 and Tenascin-C. Thus, in gingival fibroblasts GSK3α/β controls C×43 regulated expression of molecules involved in proteolytic processing of the wound ECM, cytokines and growth factors (MMP-1, TIMP-1 and -3), and ECM deposition (TGF-β1) [37,62,63].

Interestingly, Gap27 treatment also significantly increased expression of C×43 at both mRNA and total protein levels, although it did not affect relative proportions of the differently phosphorylated forms of the protein in the Western blots. Increased C×43 mRNA expression was also noted after MFA treatment. These findings are different from previous observations where treatment of human skin fibroblasts with Gap27 in a scratch wound model did not upregulate C×43 levels, but promoted its phosphorylation at S368 [97]. It is possible that Gap27 treatment of confluent cell layers (as in the present study) or in the scratch wounding protocol [97] may result to a different cell response to the peptide. Another possibility is that the responses depend on the previously described distinct phenotype of human gingival and skin fibroblasts [53,93]. In any case, in the present study, the dye transfer assays, which showed reduced GJ-mediated dye transfer by Gap27 treatment, were performed after 24 h pre-incubation with Gap27 to allow Gap27-induced C×43 upregulation to occur. Thus, despite of the elevated levels of C×43 in gingival fibroblasts, Gap27 was still able to block C×43 GJ channel functions as expected. Therefore, whether Gap27-induced gene expression changes in cultured human gingival fibroblasts depend on Gap27-induced upregulation of C×43 expression and/or reduced GJ function, and how this relates to reduced C×43 immunostaining and potential function in gingival wounds, remains to be shown.

In order to assess the mechanisms of Gap27-induced C×43 upregulation in gingival fibroblasts we used pharmacological inhibitors to key signaling pathways that associate with C×43. Previous findings have indicated that transcriptional modulator AP1 regulates C×43 expression by various signals [5]. Accordingly, its inhibition also partially suppressed Gap27-induced C×43 expression. In addition, Gap27-induced C×43 expression was partially regulated by MEK1/2 and p38 inhibitors, two upstream modulators of AP1 [98,99]. Interestingly, though, Gap27-induced C×43 induction was totally blocked by the SP1 inhibitor, suggesting that this transcription factor is another important regulator of Gap27-induced C×43 expression in gingival fibroblasts. A recent molecular study has also linked SP1 to regulation of C×43 expression [100]. SP1 also modulates C×43-induced expression of a set of genes in various cells [73]. Accordingly, blocking of SP1 totally or partially inhibited Gap27-induced expression of 7 genes in the present study.

To summarize, our findings demonstrate that C×43 shows similar spatiotemporal regulation in gingival wound epithelium over time as previously describe for skin. In addition, we showed for the first time that the abundance of C×43-positive plaques is strongly reduced in fibroblasts at the early stages of human gingival wound healing, returning to the level of normal tissue by day 60 post-wounding. Thus, wounding-induced suppression of C×43 in wound fibroblasts leads to disruption of the connexin-mediated intercellular communication network in the connective tissue, resulting to a gene expression change that maybe important for the fast and scarless wound healing outcome in gingiva. Interestingly, blocking of C×43 function by mimetic peptides strongly regulated expression of a number of wound healing and scar formation-associated genes in human gingival fibroblasts. These changes involved p38, MEK1/2-ERK1/2, TGF-β-SMAD3 and GSK3α/β mediated signaling pathways, and AP1 and SP1 transcription factors. Among these pathways, ERK1/2-MEK1/2 appeared to be a key regulator of C×43 mimetic peptide-modulated gene expression. Thus, C×43 mimetic peptides may provide an efficient tool to modulate fibroblast gene expression during wound healing. The exact mechanisms by which the C×43 mimetic peptides cause these effects, and the mechanisms and importance of C×43 down regulation for fast and scarless wound healing outcome in human gingiva *in vivo* warrant further investigation.

## Supporting Information

S1 Fig C×43 is down regulated in gingiva during wound healing. Representative immunostainings of C×43 (red) and vimentin (green; a mesenchymal cell marker) in unwounded human oral mucosal tissue (attached gingiva) (A–C), and in gingival wounds 3- (D-F), 7- (G-I), 14- (J-L), 28- (M-O) and 60-days (P-Q) post-wounding. (A–C) In unwounded gingiva, abundant C×43 staining was localized in suprabasal epithelial cells. Most intensely stained cells were located in the stratum spinosum, but weak staining was also noted in basal epithelial cells. Inserts in (B) and (C) show higher magnification images of C×43 localization in basal cells at the connective tissue papilla and rete peg areas, respectively. (D–F) At day 3 post-wounding, C×43 was down regulated in migrating epithelial cells (D and E) and fibroblasts at wound edge (F; arrowheads indicate wound edge). (G–I) At day 7 post-wounding, when the wound was completely covered with a new epithelium, 2–3 most basal epithelial cell layers showed C×43 staining in the wound epithelium, while there was only a very weak immunoreactivity for C×43 in the spinous layer (G and H). Very little C×43 immunoreactivity was noted in fibroblasts at the wound edge (I). (J–L) At day 14 post-wounding, C×43 was confined to the 2–3 most basal layers of wound epithelium (J and K). At this stage, immunoreactivity for C×43 was slightly increased at the wound edge connective tissue (L) as compared to 7-day wounds (I). (M–O) At day 28 after wounding, C×43 immunoreactivity was normalized in the epithelium at the wound site, being present mainly in suprabasal cells of the stratum spinosum (M and N). Abundance of C×43-positive plaques in connective tissue cells at the wound edge (O) was increased as compared to earlier time points (I and L). (P–Q) At day 60 after wounding, structure of the epithelium and connective tissue formed at the wound area was closely similar to unwounded tissue. C×43 immunoreactivity was also similar to unwounded tissue in the epithelium at the wound site (P and Q). (R) Negative control staining of C×43 in unwounded epithelium. Data shown represents minimum of three sections stained in parallel samples from two to three individual donors at each time point. Arrowheads (D, E, F, G, I, J and M) indicate wound edge. E: epithelium; CT: connective tissue; FC: fibrin clot; W Edge: wound edge; WE: wound epithelium; GT: granulation tissue; WCT: wound connective tissue. Nuclear staining (blue) was performed using DAPI. Magnification bars in the inserts in B, C and Q: 50 μm.
(TIF)

S2 Fig Immunolocalization of C×43 in gingival wound macrophages. (A and B) Representative images of wound samples double immunostained with anti-C×43 (red) and anti-Clever-1 (green; M2 macrophage marker) antibodies. (A) At day 14 post-wounding, very few C×43-positive structures (arrowheads) were noted in some of the macrophages located in the newly made wound connective tissue. (B) At day 28 post-wounding, number of M2 macrophages was strongly reduced compared to day 14, with very little macrophage-associated C×43 immunoreactivity. Magnification bar: 10 μm.
(TIF)

S3 Fig Immunolocalization of C×43 in gingival wound myofibroblasts. (A and B) Representative images of the same wound location in parallel day 14 wound sections stained with an antibody against α-SMA (A) and C×43 (B). At day 14 post-wounding, the wound contained numerous α-SMA-positive myofibroblasts. However, very few C×43-positive structures (arrowheads) were noted in cells in the myofibroblast-rich area. Magnification bar: 10 μm.
(TIF)

S4 Fig Phase contrast images of gingival fibroblast cultures treated with or without Gap27. Confluent fibroblast cultures (GFBL-DC) were cultured in their normal growth medium (DMEM) (A), or treated with control peptide (B) or Gap27 (C) (150 μM), and images acquired 24 h after treatment. Magnification bar: 50 μm.
(TIF)

S5 Fig Gap27-treatment promotes gingival fibroblast migration. (A) Representative images of human gingival fibroblast (GFBL-DC) migration in the presence of Gap27 or control peptide (150 μM) across a scrape wound over time. Lines indicate original wound margins. Magnification bar: 20 μm. (B) Quantification of Gap-27-induced fibroblast migration over time. (C) Representative images of human gingival fibroblast (GFBL-DC) migration in the presence of control siRNA-1 or -2 or C×43 siRNA-1 or -2 (30 nM) across a scrape wound over time. Wounds were completely closed in all groups at 24 h. Lines indicate original wound margins. Magnification bar: 40 μm. (D) Quantification of cell migration in C×43 and control siRNA treated samples over time. Results show pooled data for C×43 siRNA-1 and -2, and control siRNA-1 and -2 treated samples, respectively. Wounds were completely closed in all groups at 24 h. For the experiments, siRNA transfection was performed 30 h before wounding. Wound closure rate was determined measuring the area of the open wound at each time point relative to the area of the same wound at the time of wounding. Results show mean +/− SEM from minimum of triplicate samples. Statistical testing was performed comparing test and control samples at the given time point (*p<0.05, **p<0.01; Student’s t-test). Non-treated samples (incubated in DMEM only) did not show difference to control peptide or control siRNA-treated samples, and are not shown.
(TIF)

S6 Fig Effect of Gap26 and Gap27 treatment on gene expression in gingival fibroblasts. Confluent fibroblast cultures (GFBL-DC) were treated with Gap26 or control peptide (300 μM), and Gap27 or control peptide (150 μM) for 24 h, and expression of a set of genes was analyzed by real-time PCR. Results show mean mRNA expression relative to control-peptide treated samples from triplicate samples from one experiment. Horizontal line indicates relative mRNA expression for the control-peptide treated samples. EDA-FN: Extra Domain A-Fibronectin; EDB-FN: Extra Domain B-Fibronectin; TN-C: Tenascin-C; α-SMA: α-Smooth Muscle Actin; VEGF-A: Vascular Endothelial Growth Factor-A.
(TIF)

S7 Fig Expression of a set of genes in gingival fibroblasts treated with connexin inhibitor meclofenamic acid (MFA) relative to untreated samples. Real-time PCR results from GFBL-DC cultures treated with increasing concentrations of MFA for 24 h relative to vehicle-treated samples are shown. Results represent mean of triplicate samples in one experiment. MFA induced a concentration-dependent increase in expression of MMP-1, MMP-10, Vascular Endothelial Growth Factor-A (VEGF-A), Tenascin-C (TN-C) and C×43, and down regulation of CXCL12 (SDF-1α).
(TIF)

S8 Fig C×43 siRNA treatment suppresses GJ-mediated dye transfer in gingival fibroblasts. Confluent GFBL-DC cultures transfected with control siRNA-1 (A and B), C×43 siRNA-1 (C) or C×43 siRNA-2 (D) were scrape-loaded with Lucifer Yellow (green), and dye transfer was followed for 5 min. Treatment of cells with C×43 siRNA-1 and -2 reduced markedly dye transfer as compared to control siRNA-1 (results for control siRNA-2 were identical to control siRNA-1, and are not shown). Results show representative images from triplicate samples. For the experiments, siRNA transfections were performed 48 h before the experiment. Magnification bars: 50 μm.
(TIF)

S9 Fig Modulation of Gap27-regulated gene expression in gingival fibroblasts by pharmacological inhibitors of AP1, SP1, TGF-βp MEK1/2 and GSK3α̃β signaling pathways. Confluent cultures of gingival fibroblasts (GFBL-DC) were treated with Gap27 (150 μM) with or without curcumin (AP1 inhibitor), WP631 (SP1 inhibitor), SB431542 (TGF-β inhibitor), PD184352 (p38 inhibitor), SB203580 (MEK1/2 inhibitor) or SB415286 (GSK3α/β inhibitor) for 24 h, and expression of MMPs and TIMPs (A), ECM proteins and contractility-associated genes (B), TGF-β1 and growth factors (C), and cell-cell junction proteins (D) was analyzed by real-time PCR. Results represent mean mRNA expression relative to non-treated cells from triplicate samples in one experiment. DMSO: Cells treated with the vehicle (DMSO) only; TN-C: Tenascin-C; DCN: Decorin; FMOD: Fibromodulin; α-SMA: α-Smooth Muscle Actin; NMMIIB: Non-Muscle Myosin IIB; VEGF-A: Vascular Endothelial Growth Factor-A.
(TIF)

S1 Table List of the human gingival fibroblast lines used for the study. (DOCX)

S2 Table Primers used for real-time PCR. (DOCX)

S3 Table List of antibodies used for immunostaining and Western blotting. (DOCX)
