# Supplementary figures and images for: Expression and Function of Connexin 43 in Human Gingival Wound Healing and Fibroblasts
Source: PLoS One. 2015 Jan 13;10(1):e0115524. doi: 10.1371/journal.pone.0115524 (PMC4293150; doi:10.1371/journal.pone.0115524)

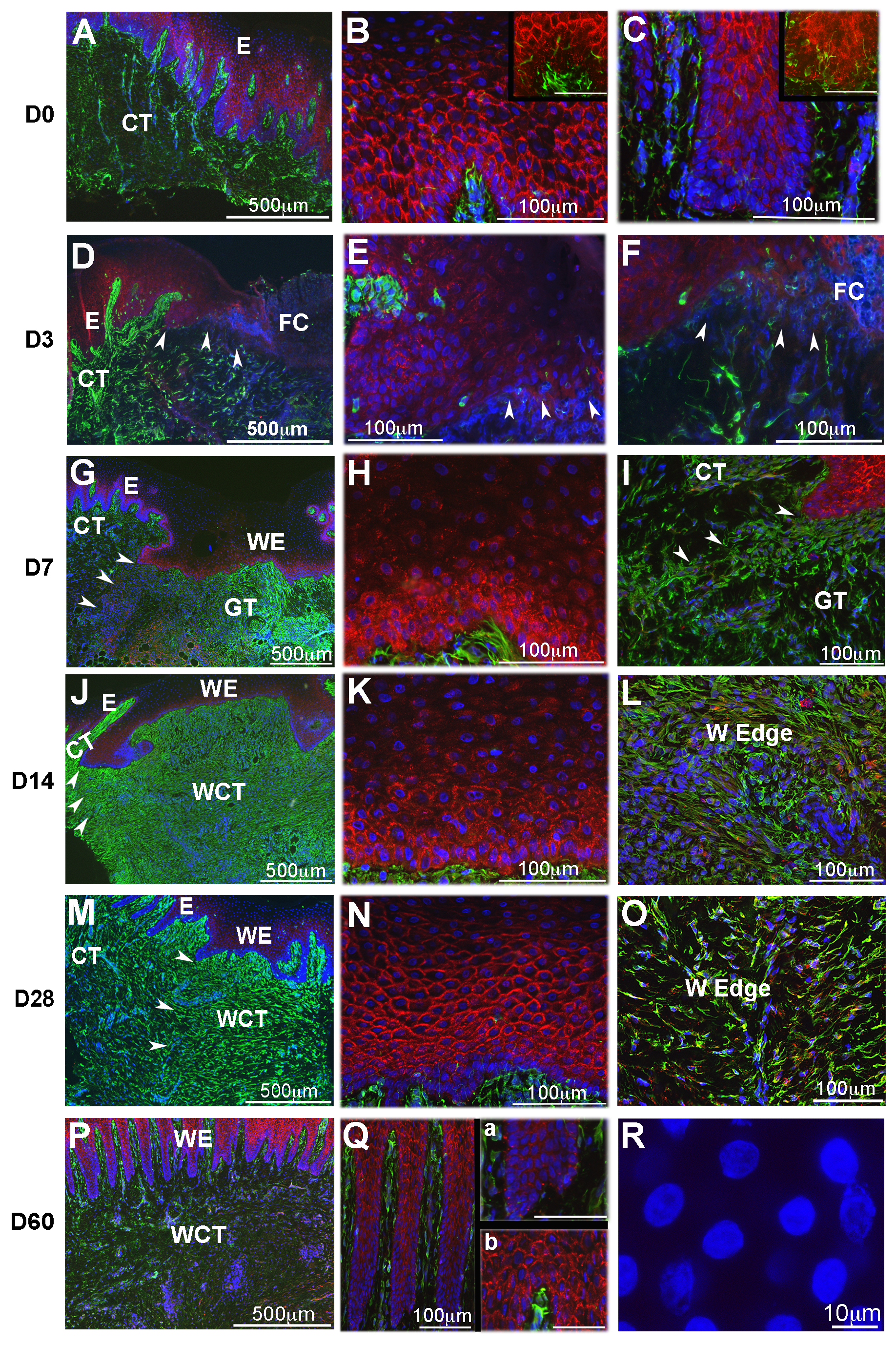

Supplement: S1 Fig — Representative immunostainings of C×43 (red) and vimentin (green; a mesenchymal cell marker) in unwounded human oral mucosal tissue (attached gingiva) (A–C), and in gingival wounds 3- (D-F), 7- (G-I), 14- (J-L), 28- (M-O) and 60-days (P-Q) post-wounding. (A–C) In unwounded gingiva, abundant C×43 staining was localized in suprabasal epithelial cells. Most intensely stained cells were located in the stratum spinosum, but weak staining was also noted in basal epithelial cells. Inserts in (B) and (C) show higher magnification images of C×43 localization in basal cells at the connective tissue papilla and rete peg areas, respectively. (D–F) At day 3 post-wounding, C×43 was down regulated in migrating epithelial cells (D and E) and fibroblasts at wound edge (F; arrowheads indicate wound edge). (G–I) At day 7 post-wounding, when the wound was completely covered with a new epithelium, 2–3 most basal epithelial cell layers showed C×43 staining in the wound epithelium, while there was only a very weak immunoreactivity for C×43 in the spinous layer (G and H). Very little C×43 immunoreactivity was noted in fibroblasts at the wound edge (I). (J–L) At day 14 post-wounding, C×43 was confined to the 2–3 most basal layers of wound epithelium (J and K). At this stage, immunoreactivity for C×43 was slightly increased at the wound edge connective tissue (L) as compared to 7-day wounds (I). (M–O) At day 28 after wounding, C×43 immunoreactivity was normalized in the epithelium at the wound site, being present mainly in suprabasal cells of the stratum spinosum (M and N). Abundance of C×43-positive plaques in connective tissue cells at the wound edge (O) was increased as compared to earlier time points (I and L). (P–Q) At day 60 after wounding, structure of the epithelium and connective tissue formed at the wound area was closely similar to unwounded tissue. C×43 immunoreactivity was also similar to unwounded tissue in the epithelium at the wound site (P and Q). (R) Negative control staini [file pone.0115524.s001.tif]

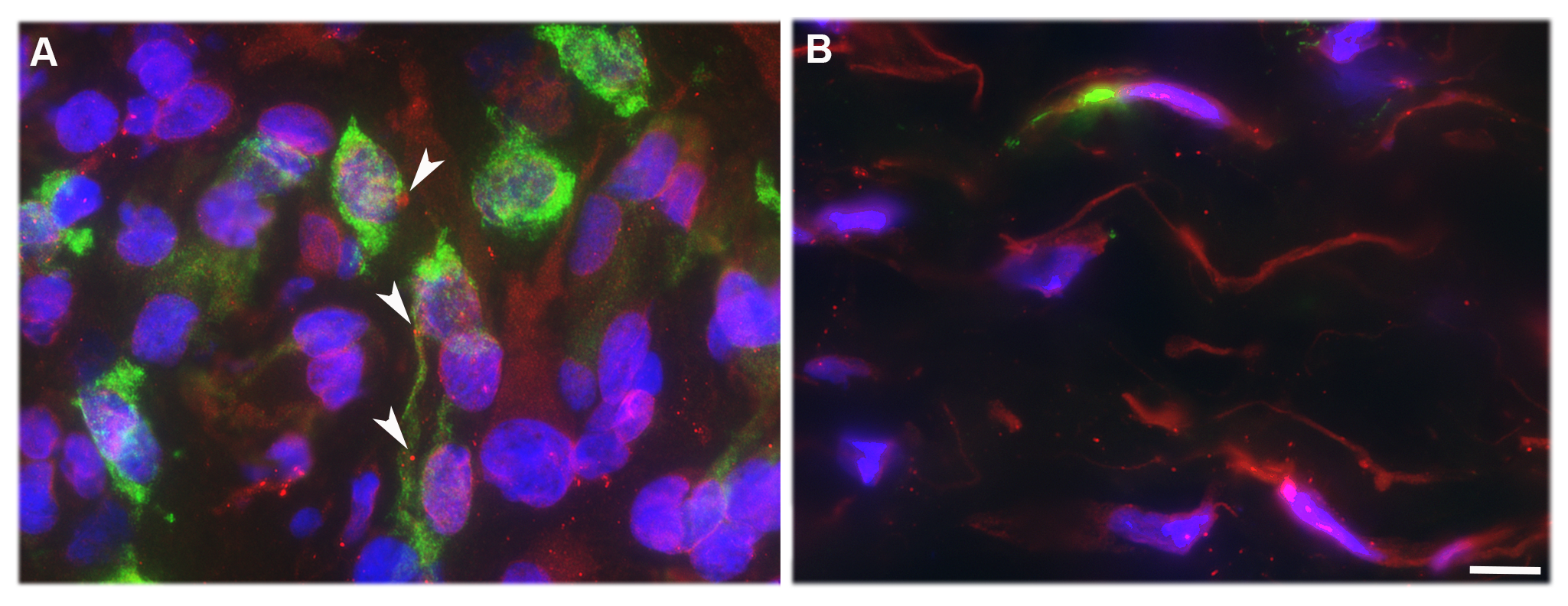

Supplement: S2 Fig — (A and B) Representative images of wound samples double immunostained with anti-C×43 (red) and anti-Clever-1 (green; M2 macrophage marker) antibodies. (A) At day 14 post-wounding, very few C×43-positive structures (arrowheads) were noted in some of the macrophages located in the newly made wound connective tissue. (B) At day 28 post-wounding, number of M2 macrophages was strongly reduced compared to day 14, with very little macrophage-associated C×43 immunoreactivity. Magnification bar: 10 μm. (TIF) [file pone.0115524.s002.tif]

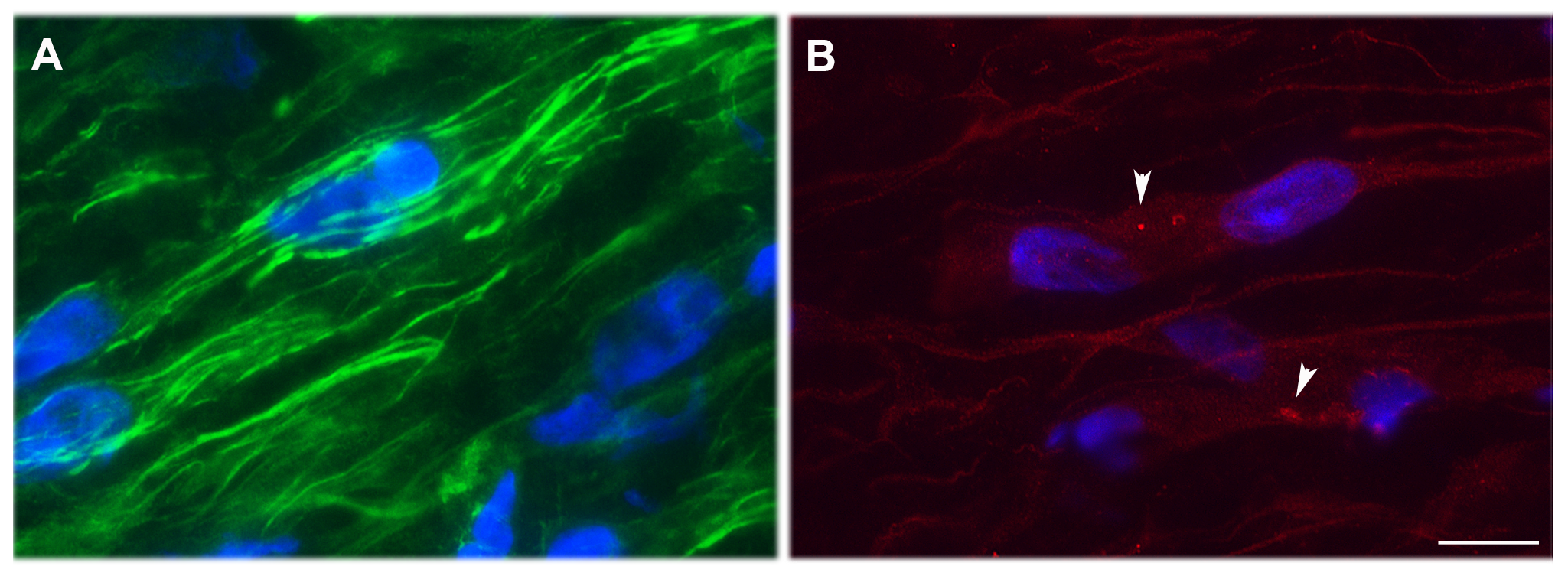

Supplement: S3 Fig — (A and B) Representative images of the same wound location in parallel day 14 wound sections stained with an antibody against α-SMA (A) and C×43 (B). At day 14 post-wounding, the wound contained numerous α-SMA-positive myofibroblasts. However, very few C×43-positive structures (arrowheads) were noted in cells in the myofibroblast-rich area. Magnification bar: 10 μm. (TIF) [file pone.0115524.s003.tif]

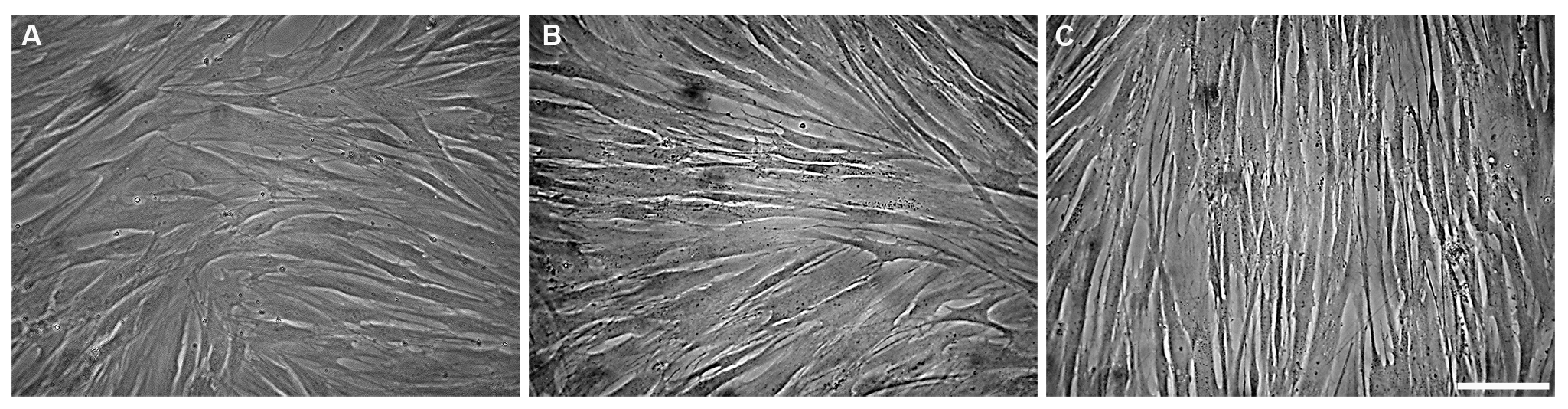

Supplement: S4 Fig — Confluent fibroblast cultures (GFBL-DC) were cultured in their normal growth medium (DMEM) (A), or treated with control peptide (B) or Gap27 (C) (150 μM), and images acquired 24 h after treatment. Magnification bar: 50 μm. (TIF) [file pone.0115524.s004.tif]

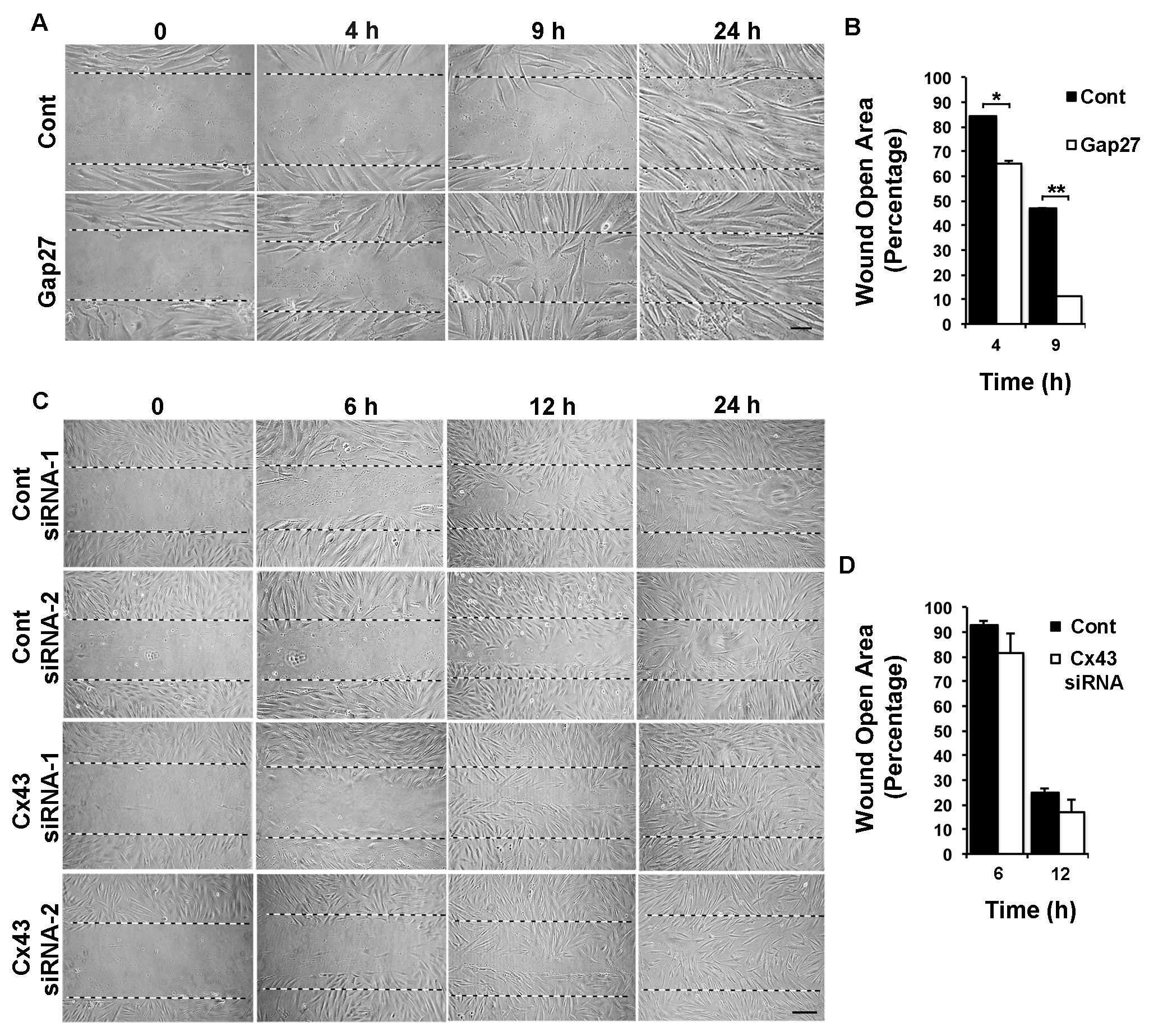

Supplement: S5 Fig — (A) Representative images of human gingival fibroblast (GFBL-DC) migration in the presence of Gap27 or control peptide (150 μM) across a scrape wound over time. Lines indicate original wound margins. Magnification bar: 20 μm. (B) Quantification of Gap-27-induced fibroblast migration over time. (C) Representative images of human gingival fibroblast (GFBL-DC) migration in the presence of control siRNA-1 or -2 or C×43 siRNA-1 or -2 (30 nM) across a scrape wound over time. Wounds were completely closed in all groups at 24 h. Lines indicate original wound margins. Magnification bar: 40 μm. (D) Quantification of cell migration in C×43 and control siRNA treated samples over time. Results show pooled data for C×43 siRNA-1 and -2, and control siRNA-1 and -2 treated samples, respectively. Wounds were completely closed in all groups at 24 h. For the experiments, siRNA transfection was performed 30 h before wounding. Wound closure rate was determined measuring the area of the open wound at each time point relative to the area of the same wound at the time of wounding. Results show mean +/− SEM from minimum of triplicate samples. Statistical testing was performed comparing test and control samples at the given time point (*p<0.05, **p<0.01; Student’s t-test). Non-treated samples (incubated in DMEM only) did not show difference to control peptide or control siRNA-treated samples, and are not shown. (TIF) [file pone.0115524.s005.tif]

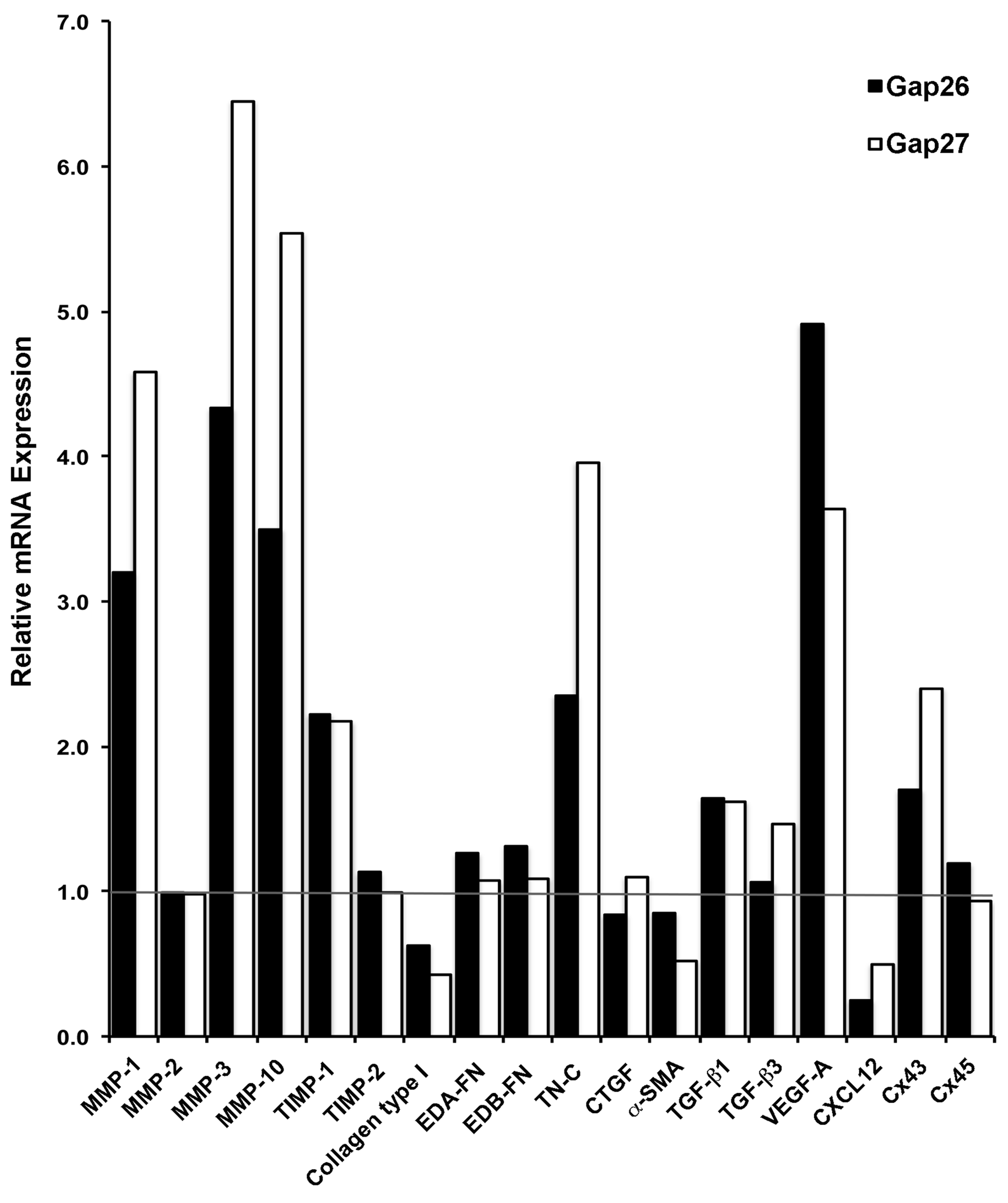

Supplement: S6 Fig — Confluent fibroblast cultures (GFBL-DC) were treated with Gap26 or control peptide (300 μM), and Gap27 or control peptide (150 μM) for 24 h, and expression of a set of genes was analyzed by real-time PCR. Results show mean mRNA expression relative to control-peptide treated samples from triplicate samples from one experiment. Horizontal line indicates relative mRNA expression for the control-peptide treated samples. EDA-FN: Extra Domain A-Fibronectin; EDB-FN: Extra Domain B-Fibronectin; TN-C: Tenascin-C; α-SMA: α-Smooth Muscle Actin; VEGF-A: Vascular Endothelial Growth Factor-A. (TIF) [file pone.0115524.s006.tif]

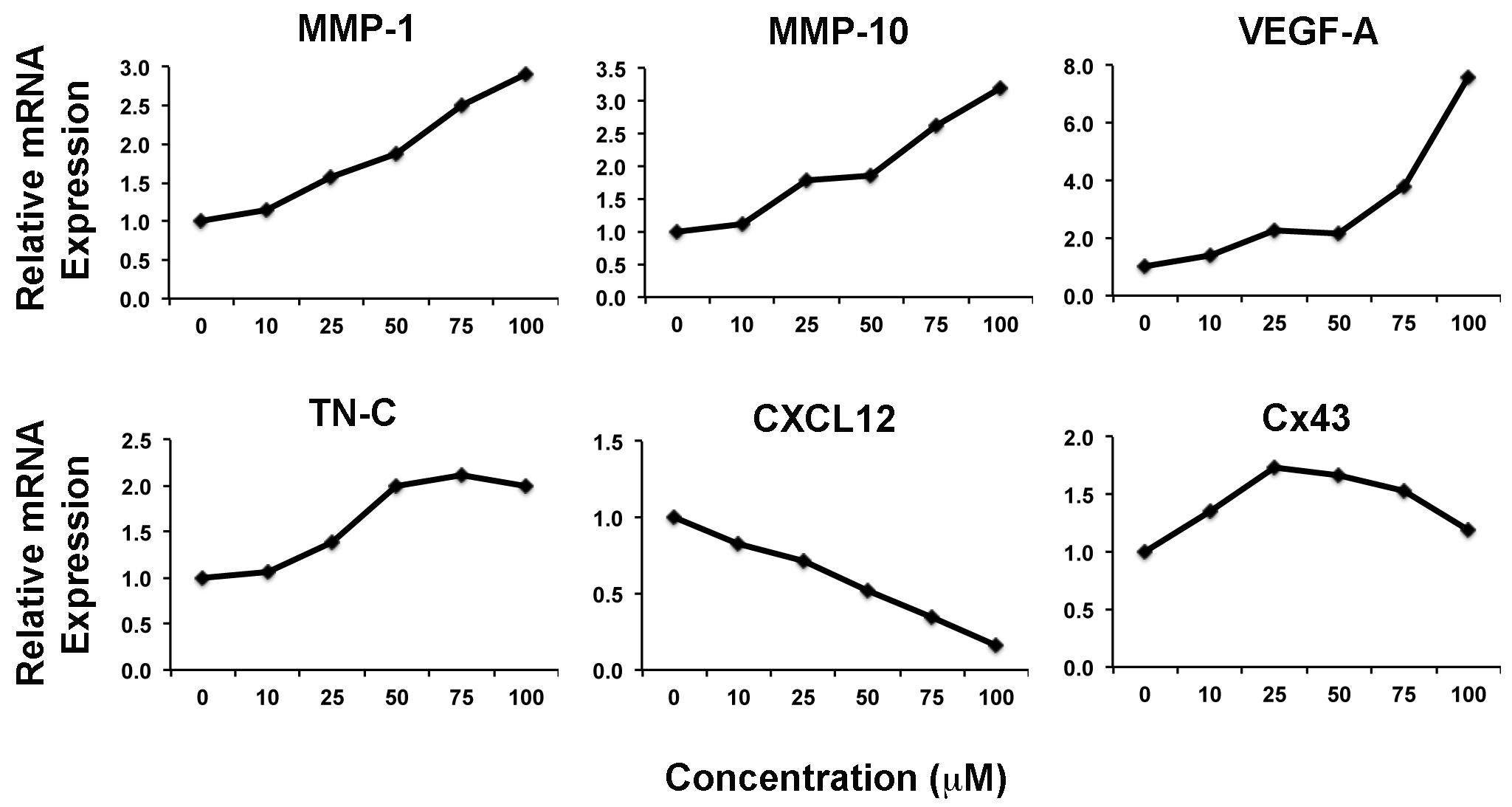

Supplement: S7 Fig — Real-time PCR results from GFBL-DC cultures treated with increasing concentrations of MFA for 24 h relative to vehicle-treated samples are shown. Results represent mean of triplicate samples in one experiment. MFA induced a concentration-dependent increase in expression of MMP-1, MMP-10, Vascular Endothelial Growth Factor-A (VEGF-A), Tenascin-C (TN-C) and C×43, and down regulation of CXCL12 (SDF-1α). (TIF) [file pone.0115524.s007.tif]

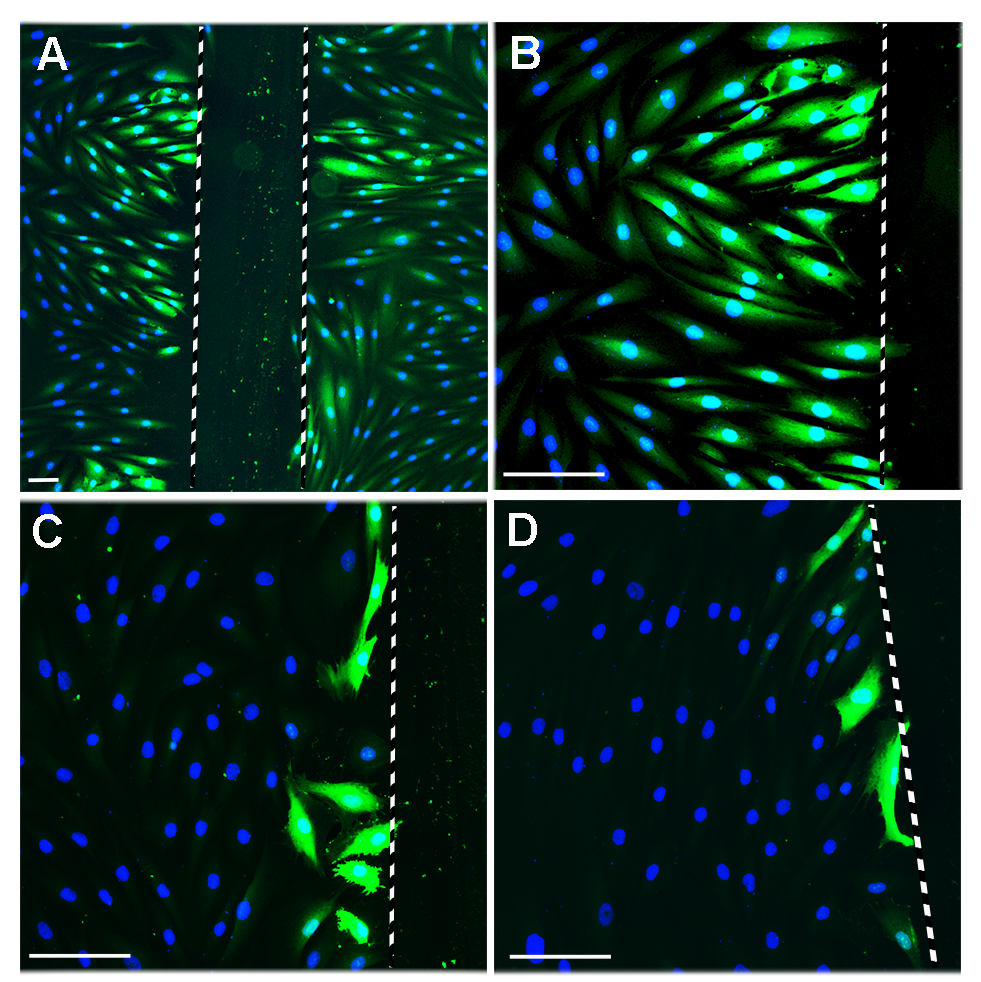

Supplement: S8 Fig — Confluent GFBL-DC cultures transfected with control siRNA-1 (A and B), C×43 siRNA-1 (C) or C×43 siRNA-2 (D) were scrape-loaded with Lucifer Yellow (green), and dye transfer was followed for 5 min. Treatment of cells with C×43 siRNA-1 and -2 reduced markedly dye transfer as compared to control siRNA-1 (results for control siRNA-2 were identical to control siRNA-1, and are not shown). Results show representative images from triplicate samples. For the experiments, siRNA transfections were performed 48 h before the experiment. Magnification bars: 50 μm. (TIF) [file pone.0115524.s008.tif]

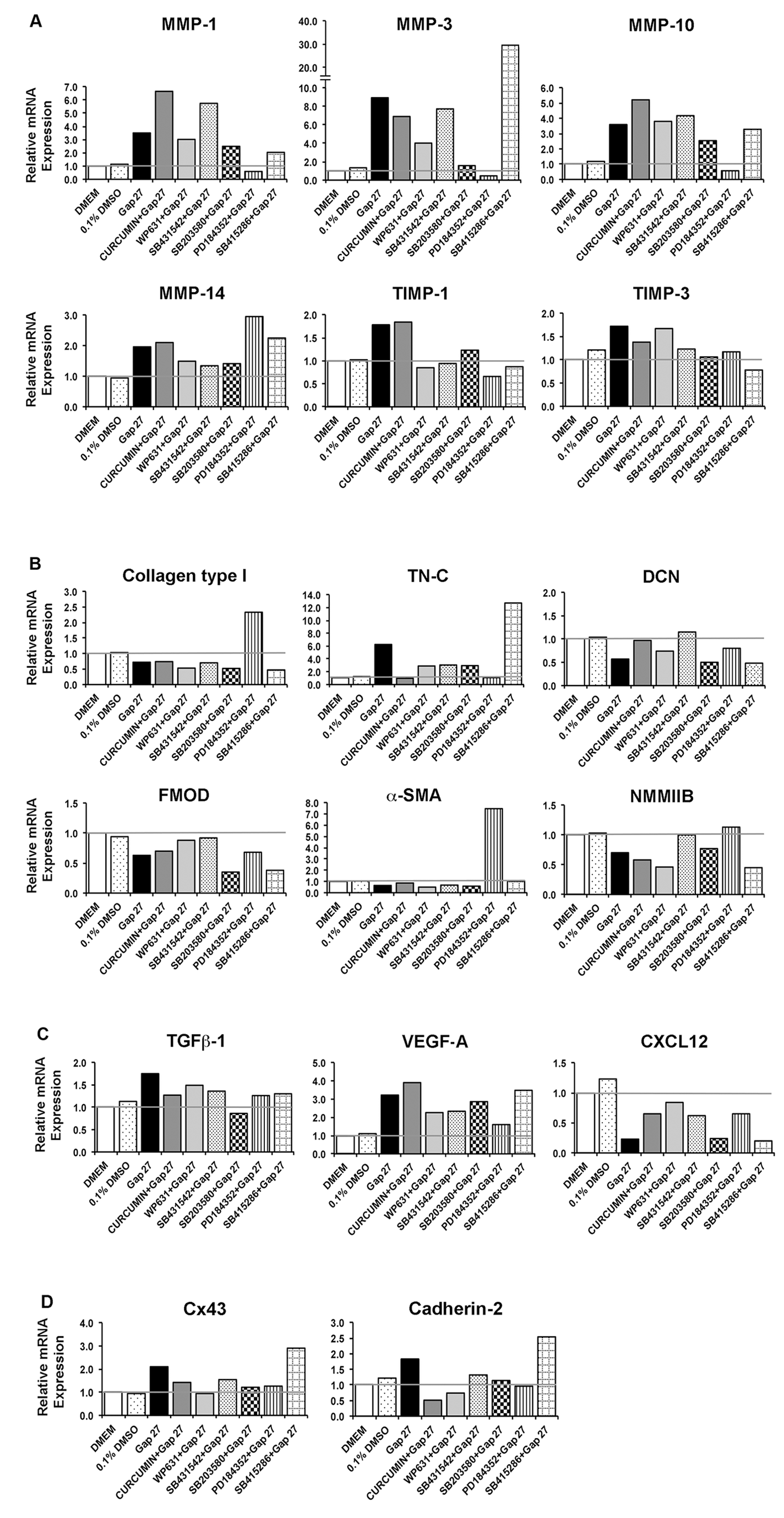

Supplement: S9 Fig — Confluent cultures of gingival fibroblasts (GFBL-DC) were treated with Gap27 (150 μM) with or without curcumin (AP1 inhibitor), WP631 (SP1 inhibitor), SB431542 (TGF-β inhibitor), PD184352 (p38 inhibitor), SB203580 (MEK1/2 inhibitor) or SB415286 (GSK3α/β inhibitor) for 24 h, and expression of MMPs and TIMPs (A), ECM proteins and contractility-associated genes (B), TGF-β1 and growth factors (C), and cell-cell junction proteins (D) was analyzed by real-time PCR. Results represent mean mRNA expression relative to non-treated cells from triplicate samples in one experiment. DMSO: Cells treated with the vehicle (DMSO) only; TN-C: Tenascin-C; DCN: Decorin; FMOD: Fibromodulin; α-SMA: α-Smooth Muscle Actin; NMMIIB: Non-Muscle Myosin IIB; VEGF-A: Vascular Endothelial Growth Factor-A. (TIF) [file pone.0115524.s009.tif]
