# Supplementary material for: Expression and Function of Connexin 43 in Human Gingival Wound Healing and Fibroblasts
Source: PLoS One. 2015 Jan 13;10(1):e0115524. doi: 10.1371/journal.pone.0115524 (PMC4293150; doi:10.1371/journal.pone.0115524)
Supplement: S1 Table — (DOCX) [file pone.0115524.s010.docx]

| **Cell line name** | **Origin** | **Sex** | **Age (years)** |
| --- | --- | --- | --- |
| GFBL-DC | Attached gingiva | Male | 41 |
| GFBL-OL | Attached gingiva | Male | 30 |
| GFBL-HN | Attached gingiva | Female | 18 |
| GFBL-DW | Attached gingiva | Female | 30 |

**Table S1. List of the human gingival fibroblast lines used for the study.**
