# Supplementary material for: Expression and Function of Connexin 43 in Human Gingival Wound Healing and Fibroblasts
Source: PLoS One. 2015 Jan 13;10(1):e0115524. doi: 10.1371/journal.pone.0115524 (PMC4293150; doi:10.1371/journal.pone.0115524)
Supplement: S3 Table — (DOCX) [file pone.0115524.s012.docx]

**Table S3. List of antibodies used for immunostaining and Western blotting.** *Non-reducing conditions were used in Western blotting.

| **Antibody** | **Manufacturer** | **Source** | **Dilution** | |
| --- | --- | --- | --- | --- |
|  |  |  | **Immunostaining** | **Western blotting** |
| Anti-Vimentin | DakoCytomation,  Burlington, ON, CA | Mouse | 1:200 |  |
| Anti-Connexin 32 | Chemicon International, Billerica, MA, USA | Mouse |  | 1:500 |
| Anti- Connexin 40 | Chemicon International | Rabbit |  | 1:1000 |
| Anti-Connexin 43 (C6219) | Sigma-Aldrich, St. Louis, MO, USA | Rabbit | 1:800 | 1:8000 |
| Anti-Connexin 45 | Santa Cruz,  Dallas, TX, USA | Rabbit | 1:500 | 1:1000 |
| Anti-MMP-1 (N-17) (sc-8834-R) | Santa Cruz | Rabbit |  | 1:1000 |
| Anti-MMP-3 (ab77962) | Abcam Inc., Cambridge, MA, USA | Mouse |  | 1:2000 |
| Anti-MMP-10 | R&D Systems Inc., Minneapolis, MN, USA | Goat |  | 1:1000 |
| Anti-VEGF (A-20)  (sc-152) | Santa Cruz | Rabbit |  | 1:500* |
| Anti-human Decorin | R&D Systems Inc. | Mouse |  | 1:500 |
| Anti-human SMAD3 (ab28379) | Abcam Inc. | Rabbit |  | 1:2000 |
| Anti-human phospho-SMAD3 (ab52903) | Abcam Inc. | Rabbit |  | 1:2000 |
| Anti-GSK3α/β (0011-A): sc-7291 | Santa Cruz | Mouse |  | 1:1000 |
| Anti- GSK3α/β (Ser21/9) | Cell Signaling, Danvers, MA, USA | Rabbit |  | 1:1000 |
| Anti-human ERK1 (ab7947) | Abcam Inc. | Rabbit |  | 1:500 |
| Anti-active MAPK  (ERK1/2) (pTEpY) | Abcam Inc. | Rabbit |  | 1:2000 |
| Anti-ACTIVE p38  (pTGpY) | Promega, Madison, WI, USA | Rabbit |  | 1:2000 |
| Anti-human p38 MAP kinase (L53F8) | Cell Signaling | Mouse |  | 1:1000 |
| Anti-active β-Catenin  (clone 8E7) | Millipore, Temecula,  CA, USA | Mouse |  | 1:1000 |
| Anti-β-Catenin (total)  (ab32572) | Abcam Inc. | Rabbit |  | 1:10000 |
| Anti-phospho-β-Catenin (Ser33/37/Thr41) | Cell Signaling | Rabbit |  | 1:1000 |
| Anti-β-Tubulin (ab21057) | Abcam Inc. | Goat |  | 1:1000 |
| Anti-α-SMA (ab5694) | Abcam Inc. | Rabbit | 1:200 |  |
| Anti-Clever-1 (M2 macrophages) | Kindly provided by Dr. Sirpa Jalkanen, University of Turku, Turk, Finland | Rat | 20 μg/ml |  |
